# Intelligent computing with Levenberg–Marquardt artificial neural network for Carbon nanotubes-water between stretchable rotating disks

**DOI:** 10.1038/s41598-023-30936-x

**Published:** 2023-03-08

**Authors:** Faizan Ali, Muhammad Awais, Aamir Ali, Narcisa Vrinceanu, Zahir Shah, Vineet Tirth

**Affiliations:** 1 Department of Mathematics, COMSATS University Islamabad, Attock Campus, Kamra Road, Attock, 43600 Pakistan; 2 Faculty of Engineering, Department of Industrial Machines and Equipments, “Lucian Blaga” University of Sibiu, 10 Victoriei Boulevard, Sibiu, Romania; 3 Department of Mathematical Sciences, University of Lakki Marwat, Lakki Marwat, Khyber Pakhtunkhwa 28420 Pakistan; 4 Mechanical Engineering Department, College of Engineering, King Khalid University, 61421 Abha, Asir Kingdom of Saudi Arabia; 5 Research Center for Advanced Materials Science (RCAMS), King Khalid University, Guraiger, P.O. Box 9004, 61413 Abha, Asir Kingdom of Saudi Arabia

**Keywords:** Mathematics and computing, Nanoscience and technology

## Abstract

Hybrid Nano fluid has emerged to be an important field of study due to its better thermal performance compared to other Nano fluids. The problem of carbon nanotubes rotating between two stretchable discs while suspended in water is investigated in this research. Due to numerous uses of this problem, such as metal mining, drawing plastic films, and cooling continuous filaments, this problem is essential to industry. Considerations here include suction/injection, heat radiation, and the Darcy-Forchheimer scheme with convective boundary conditions. The partial differential equations are reduced to ordinary differential equations by using appropriate transformation. To examine the approximate solution validation, training and testing procedures are interpreted and the performance is verified through error histogram and mean square error results. To describe the behavior of flow quantities, several tabular and graphical representations of a variety of physical characteristics of importance are presented and discussed in detail. The basic aim of this research is to examine the behaviour of carbon nanotubes (nanoparticles) between stretchable disks while considering the heat generation/absorption parameter by using the Levenberg–Marquardt technique of artificial neural network. Heat transfer rate is accelerated by a decrease in velocity and temperature and an increase in the nanoparticle volume fraction parameter which is a significant finding of the current study.

## Introduction

Carbon nanotubes are graphene sheets that have been curved into cylindrical shapes. The one graphite sheet resembles single-carbon-atom-thick chicken wire. Carbon nanotubes were first discovered by Iijima in 1991^1^, and their extraordinary mechanical, electrical, and thermal properties have sparked a flurry of activity in almost every field of area of research and engineering^2–10^. Experimental measurements have research findings show that carbon nanotubes have excellent mechanical properties, particularly in the engineering industry^11–16^. As a result, an efficient method for evaluating the fundamental properties of nano sized carbon nanotubes is required.

In the context of space technology and numerous technical applications, such as advanced types of power plants for nuclear missiles, reentry spacecraft, high-speed travels and procedures involving extreme heat, the thermal radiation effect on the free convection flow is vital. For optically thick media, the Boundary condition diffusion estimation is valid. The Discretized diffusion approximation, which resembles radiative heat fluxes for an optically thick medium, has been employed widely in various radiation studies. The wide-ranging application of heat radiation on natural convection in physics and engineering, notably in the construction of components and equipment, space technology, and gas turbines, has become increasingly relevant. England and Emery^17^ investigated the effect of heat radiation on a laminar, natural flow boundary layer which leads down a vertical plate for absorbing and non-absorbing gases. In the presence of Newtonian heating, Rajesh et al.^18^ studied the impacts of redox reactions and infrared energy on the instantaneous Magneto hydrodynamic natural convective fluid.

The study of the conduct of electrically transmitting fluids is known as magnetohydrodynamics (MHD). In 1942, Hannes Alfvén invented the term “magnetohydrodynamic.” Magnetohydrodynamics is a form of magnetohydrodynamics used in a variety of applications such as energy conversion and electric power generation. In a hybrid nanofluid, Ishak et al.^19^ presented a study on mixed convection heat transmission along a permeable stretching/shrinking curved surface. Pop et al.^20^ investigated the flow and heat transmission of hybrid nanofluids over a nonlinear porous stretching/shrinking surface.

In^21^, a mathematical method is used to model the problem of time dependent boundary layer laminar magnetized mixed convection flow of a SiO_2_-Al_2_O_3_/water hybrid nanofluid near the stagnation-point on a upright porous flat plate. To better understand how these materials perform in practical applications, the authors of^22^ probably investigate the heat transfer behaviour of the nanofluids during melting. Waqas et al.^23^ investigates the flow of viscoelastic micro polar nanoparticles presumably makes use of numerical techniques in order to better comprehend the intricate interactions between the particles and the porous media. Research paper^24^ is likely to provide a comprehensive view of the state-of-the-art in the field of hybrid nanofluids for heat transfer and to highlight areas for further research. Babu et al.^25^ summary and analysis of numerous studies on the characteristics and conduct of hybrid nanofluids and their effect on heat transfer is most likely accurate. The study^26^ focuses on how slip barriers affect the nano fluid’s flow and heat transmission characteristics.

Distortion of polymer liquids, hardening of fluid gems, and cooling of metallic plates in shower colloidal arrangement are on the whole instances of modern cycles where investigations of free convection channel stream and heat transfer attributes of couple pressure liquids are helpful. Different general speculations as well as explicit strategies have been created over the long run to depict the progression of continuum liquids. The vital element two or three stress is the execution of a size-dependent impact. Traditional continuum mechanics ignores the size impact of material particles inside the continuous. This is true even when the rotational cooperation between the liquid particles, which causes the power pressure tensor to be even, is ignored. The energy transfer caused by a convection stream in a fixed or moving chamber also numerous scientists have become interested in it because of its wide range of design and geophysical uses, including subsurface energy transit and frameworks for atomic reactor cooling. In any case, this area of investigation isn’t given the same weight as streams over a level plate, channel streams, and streams over sheets, for example. It's most likely due to the perplexing nature of these difficulties. Haq et al.^27^ studied the convective heat transfer in MHD slip flow over a stretching surface in the presence of carbon nanotubes. Fan et al.^28^ studied the mixed convection heat transfer in horizontal channel filled with nanofluids. Many other efforts in this direction are presented here^29–44^.

The objective of this research^45^ is to use an unique implementation of an intelligent numerical computing solver based on multi-layer perceptron (MLP) feed-forward back-propagation artificial neural networks (ANN) with the Levenberg-Marquard algorithm to construe the heat radiation and generation/absorption phenomena in time dependent electrically conducting Williamson fluid through a permeable extending surface. The boundary layer flow of a SWCNTS nanofluid toward three dissimilar nonlinear thin isothermal needles of paraboloid, cone, and cylinder shapes with convective boundary conditions has been predicted using an artificial neural network (ANN) in^46^. In^47^, an artificial neural network is used to study the slip and Darcy-Forchheimer phenomena for bioconvective implementations in a Powell-Eyring nanofluid model constrained by a stretching surface. The forced convective heat and mass transfer of a Nano fluid travelling radially via a thin needle was examined in study^48^ using the slip and Darcy-The Buongiorno model. Runge–Kutta fourth-order technique with shooting approach was utilized for the study.

Implementing an ANN model to assess the impact of carbon nanotubes-water between flexible rotating discs is the goal of this paper. To train, validate, and test performance evaluation indicators, three multilayer feed-forward networks with back-propagation levenberg–marquardt algorithms were used. A method of processing information that is based on modeling the human brain is called the ANN approach. It is frequently used to create forecasting and evaluation models. An ANN is made up of layers of several mutually interconnected neurons (interconnecting processing elements). Input, hidden, and output layers are the three types of layers that typically make up an ANN. Information regarding the enquiry is sent from outside to the input layer. Although they are hidden, the layer (s) do connect to other levels rather than the outer world. The outcome is sent outside by the output layer. Figure 19 depicts the feed-forward ANN structure's typical architecture. Every input neuron has a weight coefficient in the feed-forward ANN. The input signal from each neuron is calculated by multiplying those weight coefficients by the input signals and adding the results. The amount of hidden layers and hidden neurons are referred to as the network architecture. The network architecture and the quantity of inputs are directly related. It is preferable to start with one hidden layer and work your way up to two hidden layers if the first layer's performance is subpar. The power of the network will increase when the hidden layer's neurons are multiplied.

The behavior of carbon nanotubes-water between stretchable rotating discs using an artificial neural network model has not yet been studied, as far as the authors are aware. Thus, the novelty of current study centered on usefulness of artificial neural network procedure for stretchable rotating disks by taking the effects of heat generation/absorption parameter. The impacts of various significant parameters on heat flux parameter, In accordance with the outcome of the artificial neural network procedure, the Prandtl number and Hartmann number are explored, and numerical findings are supplied. A detailed research of the literature reveals that no previous studies of this kind have been conducted. This work is significant because it tries to close a gap in the body of knowledge in this area. By means of graphs and numerical benchmarks, the results are displayed and developed.

## Mathematical formulation

Consider a radiative flow of hybrid nanofluid in which carbon nanotubes are nano particles which are suspended between two rotating plates which are stretchable and held parallel to each other and water is used as a based fluid. “Radiative” is used to describe the type of flow present in the system. In this case, radiative flow refers to the flow of heat by radiation. Radiative heat transfer occurs when heat is transferred by electromagnetic waves, such as infrared radiation, without the need for a physical medium. The word “radial” likely refers to the direction of heat flow, which may occur in a radial manner away from the center of the system.

### Assumptions

The present fluid problem has the following assumptions.

1. It contains heat generation/absorption parameter.
2. Magnetic field strength is applied in both tangential and radial directions.
3. At temperatures T1 and T2, external surfaces are heated by convection.

Following are the governing equations presenting the present flow problem^49^,

1 $$\frac{\partial u}{\partial r} + \frac{\partial w}{\partial z} + \frac{u}{r} = 0,$$

2 $$u\frac{\partial u}{{\partial r}} + w\frac{\partial u}{{\partial z}} - \frac{{v^{2} }}{r} = \nu_{nf} \left( {\frac{{\partial^{2} u}}{{\partial r^{2} }} + \frac{1}{r}\frac{\partial u}{{\partial r}} + \frac{{\partial^{2} u}}{{\partial z^{2} }} - \frac{u}{{r^{2} }}} \right) - \left( {\frac{{\sigma_{nf} }}{{\rho_{nf} }}B_{0}^{2} + \frac{{\nu_{nf} }}{k}} \right)u - \frac{F}{\sqrt k }u^{2} ,$$

3 $$u\frac{\partial v}{{\partial r}} + w\frac{\partial v}{{\partial z}} - \frac{uv}{r} = \nu_{nf} \left( {\frac{{\partial^{2} v}}{{\partial r^{2} }} + \frac{1}{r}\frac{\partial v}{{\partial r}} + \frac{{\partial^{2} v}}{{\partial z^{2} }} - \frac{v}{{r^{2} }}} \right) - \frac{{\sigma_{nf} }}{{\rho_{nf} }}B_{0}^{2} v,$$

4 $$u\frac{\partial w}{{\partial r}} + w\frac{\partial w}{{\partial z}} = \nu_{nf} \left( {\frac{{\partial^{2} w}}{{\partial r^{2} }} + \frac{1}{r}\frac{\partial w}{{\partial r}} + \frac{{\partial^{2} w}}{{\partial z^{2} }}} \right) - \frac{{\nu_{nf} }}{k}w - \frac{F}{\sqrt k }w^{2} ,$$

5 $$\left( {\rho c_{p} } \right)_{nf} \left( {u\frac{\partial T}{{\partial r}} + w\frac{\partial T}{{\partial z}}} \right) = \left( {\kappa_{nf} + \frac{{16\sigma^{*} T_{2}^{3} }}{{3k^{*} }}} \right)\left( {\frac{1}{r}\frac{\partial T}{{\partial r}} + \frac{{\partial^{2} T}}{{\partial r^{2} }} + \frac{{\partial^{2} T}}{{\partial z^{2} }}} \right) + \sigma_{nf} B_{0}^{2} \left( {u^{2} + v^{2} } \right) + \frac{{Q_{0} }}{{\left( {\rho c_{p} } \right)_{nf} }}\left( {T - T_{\infty } } \right),$$

Boundary conditions:

6 $$\begin{aligned} & u = r\alpha_{1} ,\quad v = r\omega_{1} ,\quad w = W_{0} ,\quad - \kappa_{nf} \frac{\partial T}{{\partial Z}} = - h_{1} \left( {T_{1} - T} \right),\quad {\text{at }}z = 0, \\ & u = r\alpha_{2} ,\quad v = r\omega_{2} ,\quad - \kappa_{nf} \frac{\partial T}{{\partial Z}} = - h_{2} \left( {T - T_{2} } \right),\quad {\text{at }}z = h. \\ \end{aligned}$$

$\mu_{nf} ,\,\,\,\sigma_{nf} ,\,\,(\rho c_{p} )_{nf} ,\,\,\,\,\nu_{nf\,\,} \,\,and\,\,\,\,\rho_{nf}$ are the dynamic viscosity, electrical conductivity, heat capacity, kinematic viscosity and density of nanofluid respectively.

7 $$\begin{aligned} & \mu_{nf} = \frac{{\mu_{f} }}{{\left( {1 - \phi } \right)^{2.5} }},\,\,\,\nu_{nf} = \frac{{\mu_{nf} }}{{\rho_{nf} }},\,\,\,\rho_{nf} = \left( {1 - \phi } \right)\rho_{f} + \phi \rho_{CNT} , \\ & \left( {\rho c_{p} } \right)_{nf} = \left( {1 - \phi } \right)\left( {\rho c_{p} } \right)_{f} + \phi \left( {\rho c_{p} } \right)_{CNT} , \\ & \sigma_{nf} = \left( {1 + \frac{{3\left( {\frac{{\sigma_{CNT} }}{{\sigma_{f} }} - 1} \right)\phi }}{{\left( {\frac{{\sigma_{CNT} }}{{\sigma_{f} }} + 2} \right) - \left( {\frac{{\sigma_{CNT} }}{{\sigma_{f} }} - 1} \right)\phi }}} \right)\sigma_{f} , \\ & \frac{{\kappa_{nf} }}{{\kappa_{f} }} = \frac{{1 - \phi + 2\phi \frac{{\kappa_{CNT} }}{{\kappa_{CNT} - \kappa_{f} }}\ln \left( {\frac{{\kappa_{CNT} + \kappa_{f} }}{{2\kappa_{f} }}} \right)}}{{1 - \phi + 2\phi \frac{{\kappa_{f} }}{{\kappa_{CNT} - \kappa_{f} }}\ln \left( {\frac{{\kappa_{CNT} + \kappa_{f} }}{{2\kappa_{f} }}} \right)}}. \\ \end{aligned}$$

### Geometry

**Figure 1:**
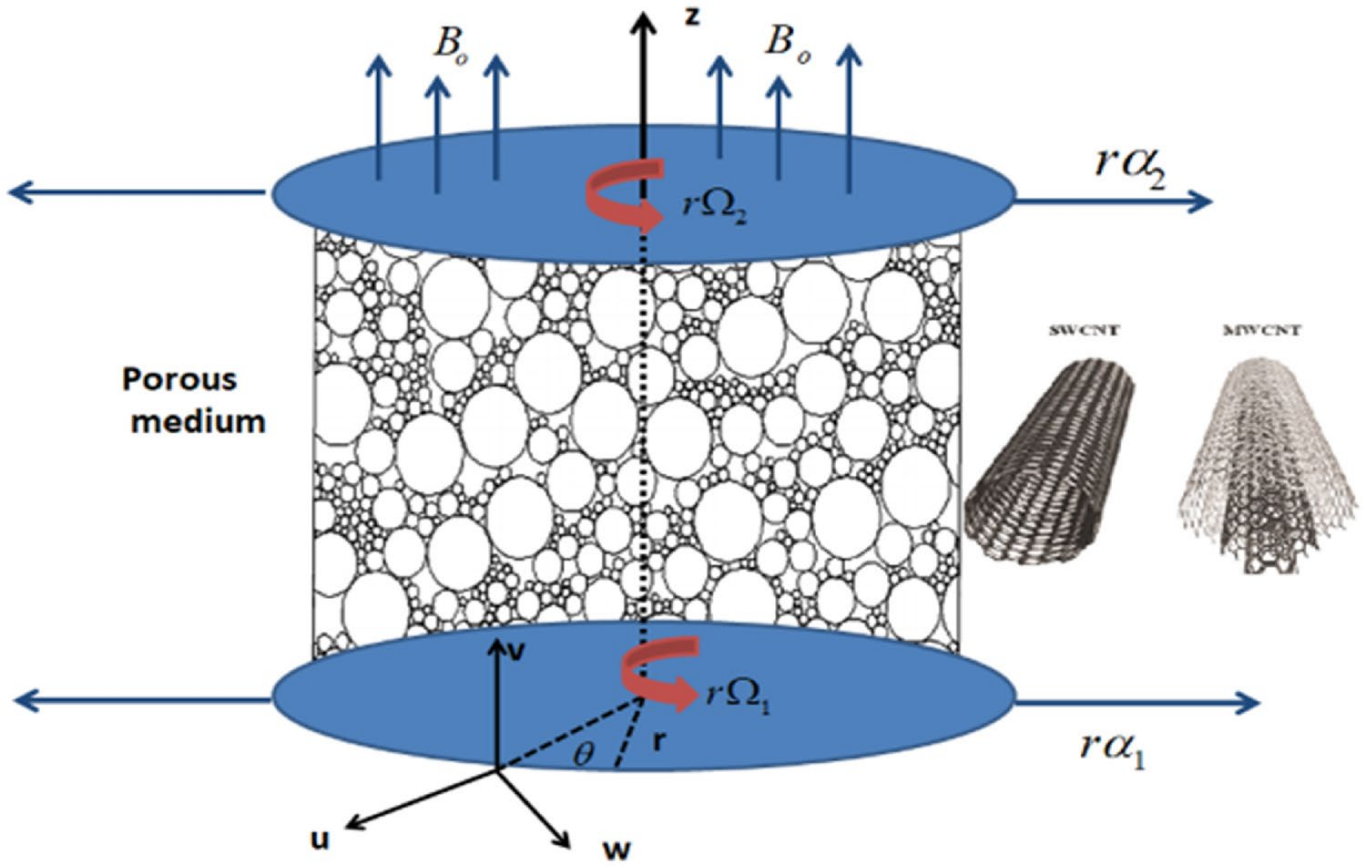
Geometry of the problem.

Figure 1, shows the physical illustration of current flow problem in which fluid containing SWCNT and MWCNT as nanoparticles between two rotating disks is observed.

Thermo-physical properties of the nanoparticles are presented in Table 1.

**Table 1:** Thermo-physical properties of H_2_O and CNTs^44,45^.

Physical properties	$c_{p} \left( {{\text{J}}/{\text{kg}}\,{\text{K}}} \right)$	$\rho (kg/m^{3} )$	$\kappa (W/m\,K)$	$\sigma (s/m)$
$H_{2} O$	4179	997.1	0.613	0.05
SWCNTs	425	2600	6600	10^6^–10^7^
MWCNTs	796	1600	3000	10^6^–10^7^

### Similarity transformations

Define the following similarity transformations:

8 $$u = r\omega_{1} ,\quad v = r\omega_{1} g\left( \eta \right),\quad w = - 2h\omega_{1} f\left( \eta \right),\quad \theta = \frac{{T - T_{2} }}{{T_{1} - T_{2} }},\quad \eta = \frac{z}{h}.$$

Equations (2)–(6) after similarity transformation becomes, and Eq. (1) is satisfied:

9 $$u = r\omega_{1} ,\quad v = r\omega_{1} g\left( \eta \right),\quad w = - 2h\omega_{1} f\left( \eta \right),\quad \theta = \frac{{T - T_{2} }}{{T_{1} - T_{2} }},\quad \eta = \frac{z}{h}.$$

10 $$\frac{1}{{\left( {1 - \phi } \right)^{2.5} \left( {1 - \phi + \phi \frac{{\rho_{CNT} }}{{\rho_{f} }}} \right)}}f^{\prime \prime \prime \prime } + {\text{R}}_{e} \left( \begin{gathered} 2ff^{\prime\prime} - f^{\prime 2} + g^{2} - \frac{{\sigma_{nf} }}{{\sigma_{f} }}\frac{Ha}{{\left( {1 - \phi + \phi \frac{{\rho_{CNT} }}{{\rho_{f} }}} \right)}}f^{\prime } \hfill \\ - \frac{\lambda }{{(1 - \phi )^{2.5} \left( {1 - \phi + \phi \frac{{\rho_{CNT} }}{{\rho_{f} }}} \right)}}f^{\prime } - F_{r} f^{\prime 2} \hfill \\ \end{gathered} \right) = 0,$$

11 $$\frac{1}{{(1 - \phi )^{2.5} \left( {1 - \phi + \phi \frac{{\rho_{CNT} }}{{\rho_{f} }}} \right)}}g^{\prime \prime } + {\text{R}}_{e} \left( {2fg^{\prime } - 2gf^{\prime } - \frac{{\sigma_{nf} }}{{\sigma_{f} }}\frac{Ha}{{\left( {1 - \phi + \phi \frac{{\rho_{CNT} }}{{\rho_{f} }}} \right)}}g} \right) = 0,$$

12 $${\text{R}}_{e} \left( {\frac{2\lambda }{{(1 - \phi )^{2.5} \left( {1 - \phi + \phi \frac{{\rho_{CNT} }}{{\rho_{f} }}} \right)}}f - 4ff^{\prime } - 4F_{r} f^{2} } \right) - \frac{2}{{(1 - \phi )^{2.5} \left( {1 - \phi + \phi \frac{{\rho_{CNT} }}{{\rho_{f} }}} \right)}}f^{\prime \prime } = 0,$$

13 $$\frac{1}{{{\text{P}}_{r} }}\left( {\frac{{\kappa_{nf} }}{{\kappa_{f} }} + R_{d} } \right)\theta^{\prime \prime } + 2\,{\text{R}}_{e} \left( {1 - \phi + \phi \frac{{\left( {\rho c_{p} } \right)_{CNT} }}{{\left( {\rho c_{p} } \right)_{f} }}} \right)f\theta^{\prime } + \frac{{\sigma_{nf} }}{{\sigma_{f} }}{\text{R}}_{e}\, HaE_{c} \left( {f^{\prime 2} + g^{2} } \right) + Q\theta = 0.$$

After similarity transformation boundary conditions takes the form.

14 $$\begin{gathered} f(0) = ws,\quad f^{\prime } (0) = A_{1} ,\quad f^{\prime } (1) = A_{2} ,\quad g(0) = 1,\quad g(1) = \omega , \hfill \\ \theta^{\prime } (0) = - \frac{{\kappa_{f} }}{{\kappa_{nf} }}\gamma_{1} (1 - \theta (0)),\quad \theta^{\prime } (1) = - \frac{{\kappa_{f} }}{{\kappa_{nf} }}\gamma_{2} \theta (1). \hfill \\ \end{gathered}$$

For lower and upper stretching plates $\gamma_{1}$ and $\gamma_{2}$ are the Biot parameters, $A_{1} \,\,and\,\,A_{2}$ are the stretching parameters, $\omega$ is the rotational parameter, $E_{c}$ is the Eckert number, $F$ is the local inertial parameter while suction/ injection parameter, Hartmann coefficient, radiation parameter, Reynolds number and porosity coefficient are presented by $ws,\,\,Ha,\,\,R_{d} ,\,\,{\text{R}}_{e} ,\,\,and\,\,\lambda$ respectively are defined by:

15 $$\begin{aligned} & \gamma_{1} = \frac{{h_{1} h}}{{\kappa_{f} }},\,\,\,\,\gamma_{2} = \frac{{h_{2} h}}{{\kappa_{f} }},\,\,\,\,A_{1} = \frac{{\alpha_{1} }}{{\omega_{1} }},\,\,\,\,A_{2} = \frac{{\alpha_{2} }}{{\omega_{2} }},\,\,\,\,\omega = \frac{{\omega_{2} }}{{\omega_{1} }}, \\ & E_{c} = \frac{{r^{2} \omega_{1}^{2} }}{{c_{p} (T_{1} - T_{2} )}},\,\,\,\,Fr = \frac{F}{\sqrt K },\,\,\,\,ws = \frac{{ - w_{o} }}{{2h\omega_{1} }},\,\,\,\,Ha = \frac{{B_{o}^{2} \sigma_{f} }}{{\rho_{f} \omega_{1} }}, \\ & R_{d} = \frac{{16\sigma^{*} T_{2}^{3} }}{{3\kappa_{f} k^{*} }},\,\,\,\,{\text{R}}_{e} = \frac{{\omega_{1} h^{2} }}{{\nu_{f} }},\,\,\,\,\lambda = \frac{{\nu_{f} }}{{k\omega_{1} }},\,\,\,\,Q = \frac{{Q_{o} }}{{(C_{p} \rho )}}. \\ \end{aligned}$$

## Solution methodology

Intelligent Bayesian regularization neural network approach is exploited for solution of hybrid nano-material of carbon nanotubes (CNT’s) based fluidic system in Eqs. (10)–(13). In ANNs-BRS (Artificial neural network-Bayesian regularization scheme) 15% data is utilized for testing, 5% for validation and 80% for training. Whereas suggested ANNs-BRS architecture diagram is presented in Fig. 3. Reference dataset for hybrid nano-material carbon nanotubes based fluidic system is numerically computed by exploiting “Adams” method with the assistance of Mathematica's built-in command “NDSolve”. Table 1 shows the thermo-physical properties of $H_{2} O$ and CNTs. The dataset is generated for each involved parameter with the variation of *A1, A2, φ, F*_*r*_*, ws, Q* as presented in Table 2. Later on, ANNs-BRS scheme is presented by utilizing the “nftool” function to access the graphical user interface of the neural network tool box in the Matlab environment. utilizing a target and arbitrary input values, 80 percent of the data is used for training, 15% for testing and 5% for validation. Neural network weight adjustment is done by employing the ANNs-BRS technique.

**Table 2:** Case settings and scenario analysis for the hybrid Nano fluid flow model.

Scenario	Case	Physical parameters of interest					
		$A1$	$A2$	$\varphi$	$F_{r}$	$ws$	$Q$
1	1	0.1	0.5	0.2	0.5	1.0	1.0
	2	0.2	0.5	0.2	0.5	1.0	1.0
	3	0.3	0.5	0.2	0.5	1.0	1.0
	4	0.4	0.5	0.2	0.5	1.0	1.0
	5	0.5	0.5	0.2	0.5	1.0	1.0
2	1	1.0	0.1	0.2	0.5	0.5	1.0
	2	1.0	0.2	0.2	0.5	0.5	1.0
	3	1.0	0.3	0.2	0.5	0.5	1.0
	4	1.0	0.4	0.2	0.5	0.5	1.0
	5	1.0	0.5	0.2	0.5	0.5	1.0
3	1	1.0	0.5	0.1	0.5	0.5	1.0
	2	1.0	0.5	0.2	0.5	0.5	1.0
	3	1.0	0.5	0.3	0.5	0.5	1.0
	4	1.0	0.5	0.4	0.5	0.5	1.0
	5	1.0	0.5	0.5	0.5	0.5	1.0
4	1	1.0	0.5	0.2	0.0	0.5	1.0
	2	1.0	0.5	0.2	0.5	0.5	1.0
	3	1.0	0.5	0.2	1.0	0.5	1.0
	4	1.0	0.5	0.2	1.5	0.5	1.0
	5	1.0	0.5	0.2	2.0	0.5	1.0
5	1	1.0	0.5	0.2	0.5	0.5	1.0
	2	1.0	0.5	0.2	0.5	0.5	1.0
	3	1.0	0.5	0.2	0.5	1.0	1.0
	4	1.0	0.5	0.2	0.5	1.5	1.0
	5	1.0	0.5	0.2	0.5	2.0	1.0
6	1	1.0	0.5	0.2	0.5	0.5	0.0
	2	1.0	0.5	0.2	0.5	0.5	0.5
	3	1.0	0.5	0.2	0.5	0.5	1.0
	4	1.0	0.5	0.2	0.5	0.5	1.5
	5	1.0	0.5	0.2	0.5	0.5	2.0

The mathematical equations for hybrid Nano-material of carbon nanotubes based fluidic system (6–7) for the case 4 of scenario 1 by utilizing the parameter values listed in Table 2 can be presented as follows:

## Analysis and results interpretation

Bayesian regularization backpropagation perform by a function in matlab program called “trainbr” function. This function disables validation stops by default but the reason for this is that validation is usually used a form of regularization also “trainbr” has its own form of validation built into the algorithm.

Performance analysis for hybrid Nano-material single and multiwall carbon nanotubes based fluidic system along with five cases and five scenarios namely $A1,\,\,A2,\,\,\varphi ,\,\,F_{r} ,\,ws\,\,and\,\,Q$ for trustworthy prediction of plots of temperature and velocity are shown in Figs 4, 5, 6 and 7.

**Figure 2:**
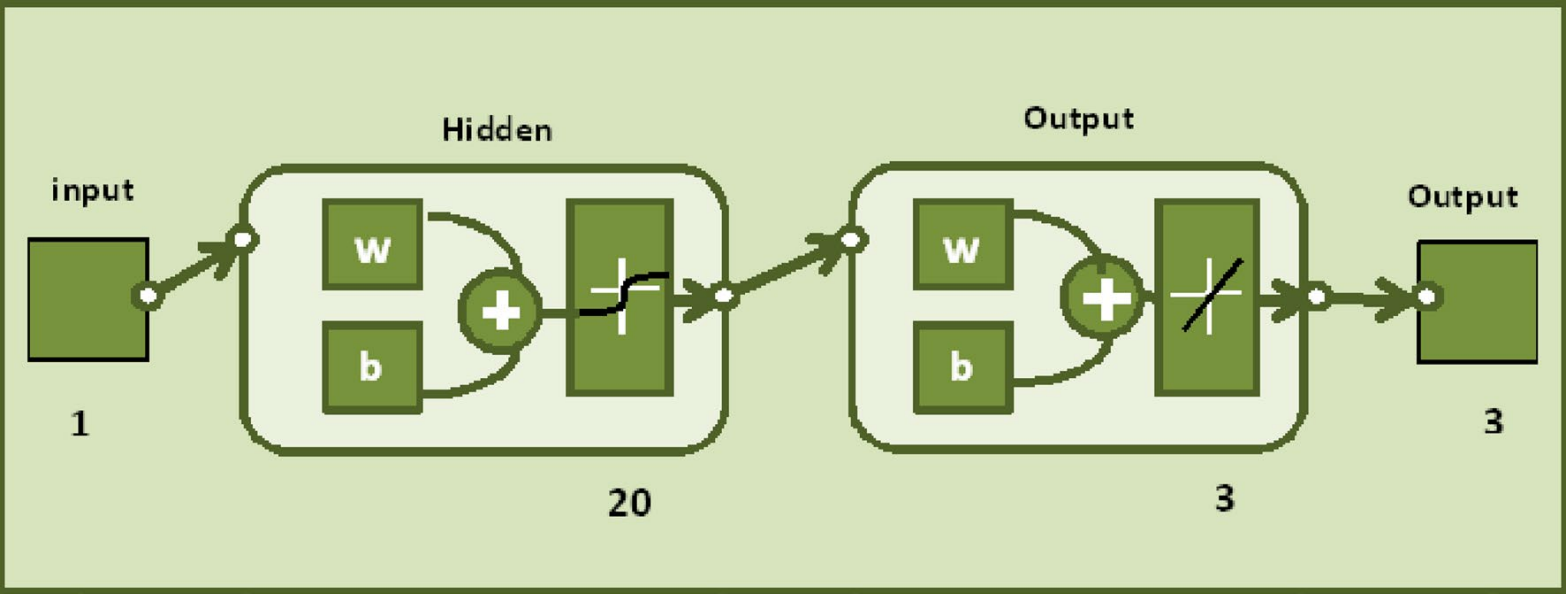
Networks architecture of ANNs-BRS scheme.

**Figure 3:**
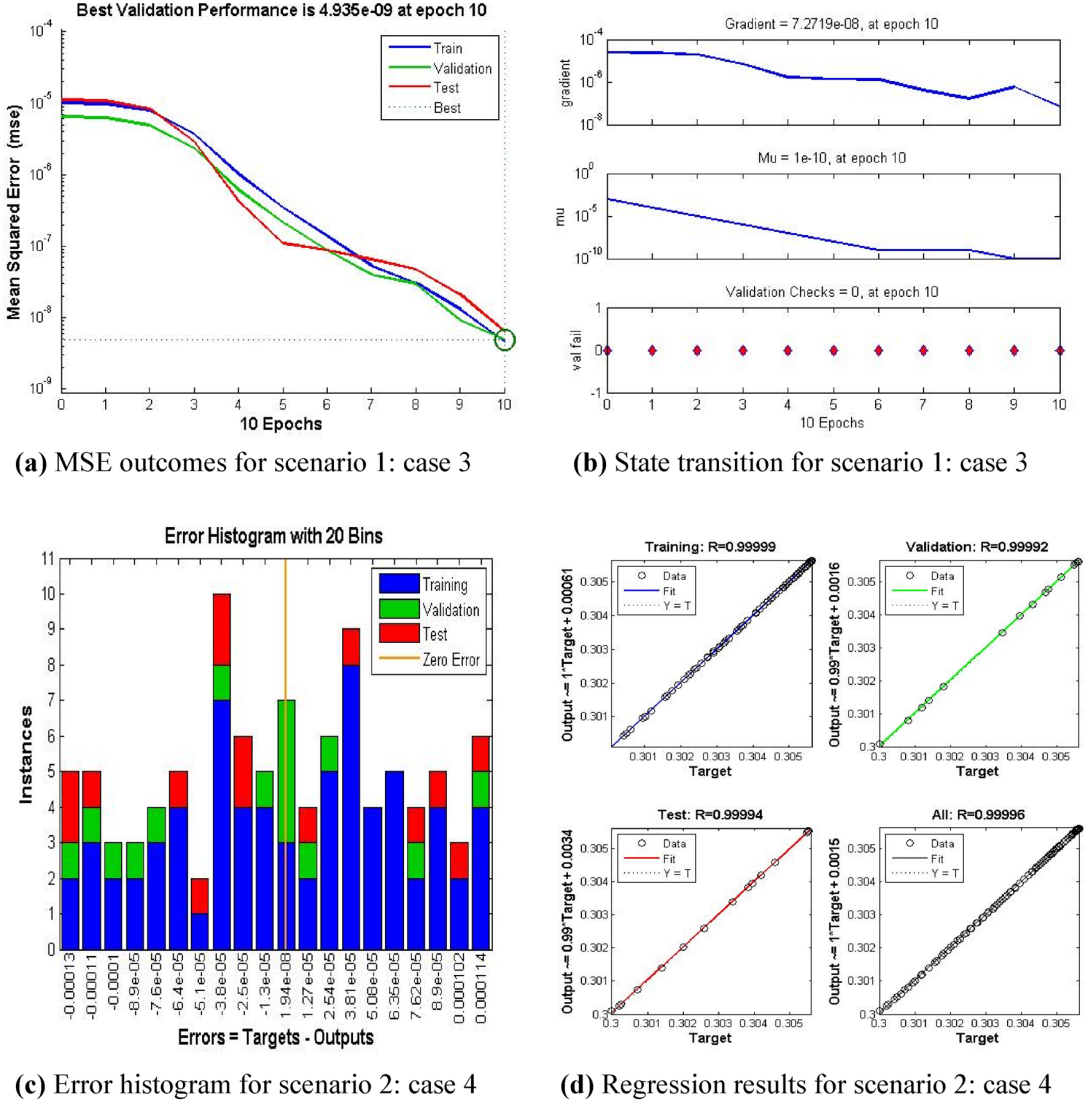
ANNs-BRS outcomes for solving the hybrid nanofluid flow model in scenario 1 case 3.

**Figure 4:**
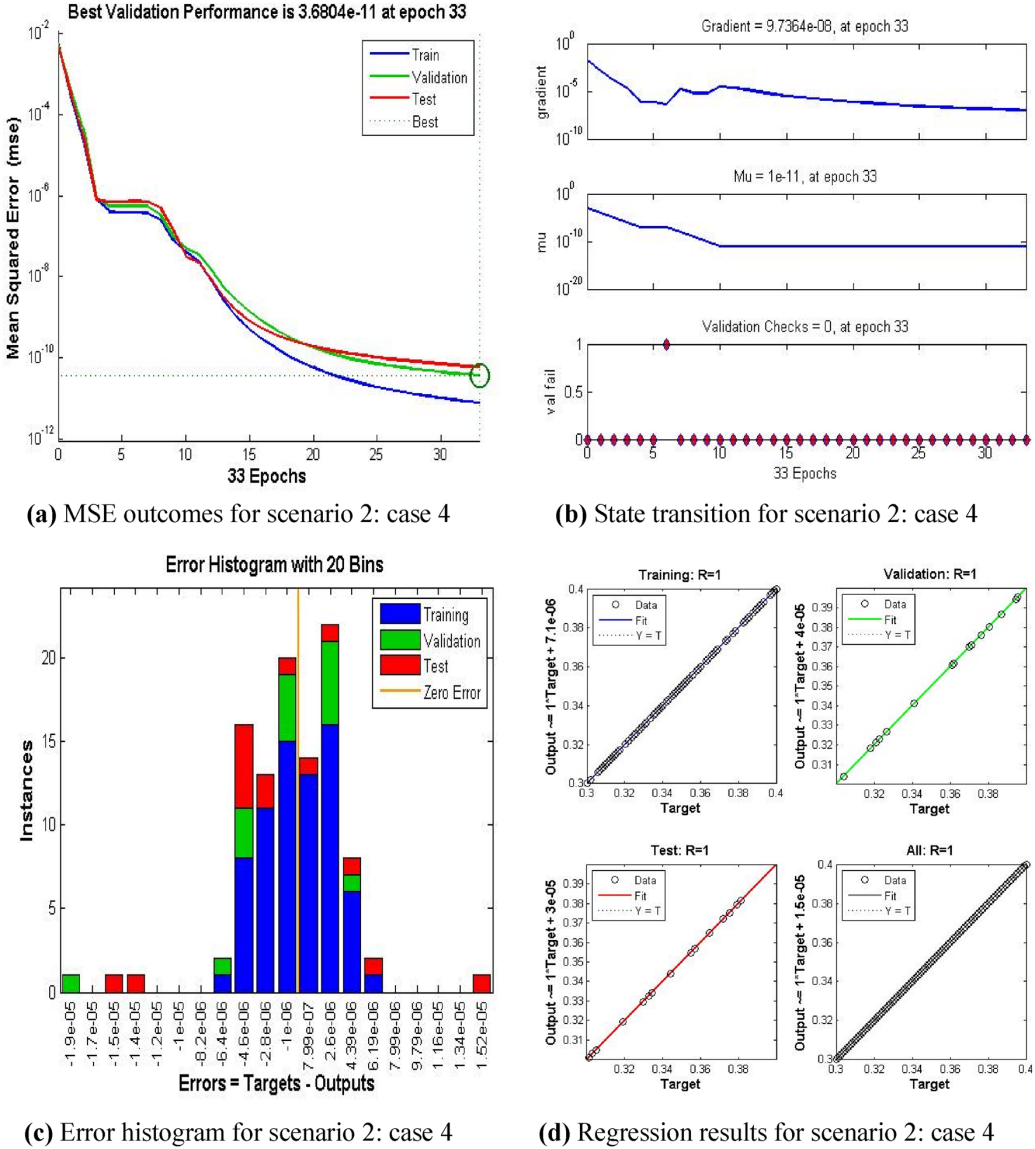
ANNs-BRS outcomes for solving the hybrid nanofluid flow model in scenario 2 case 4.

**Figure 5:**
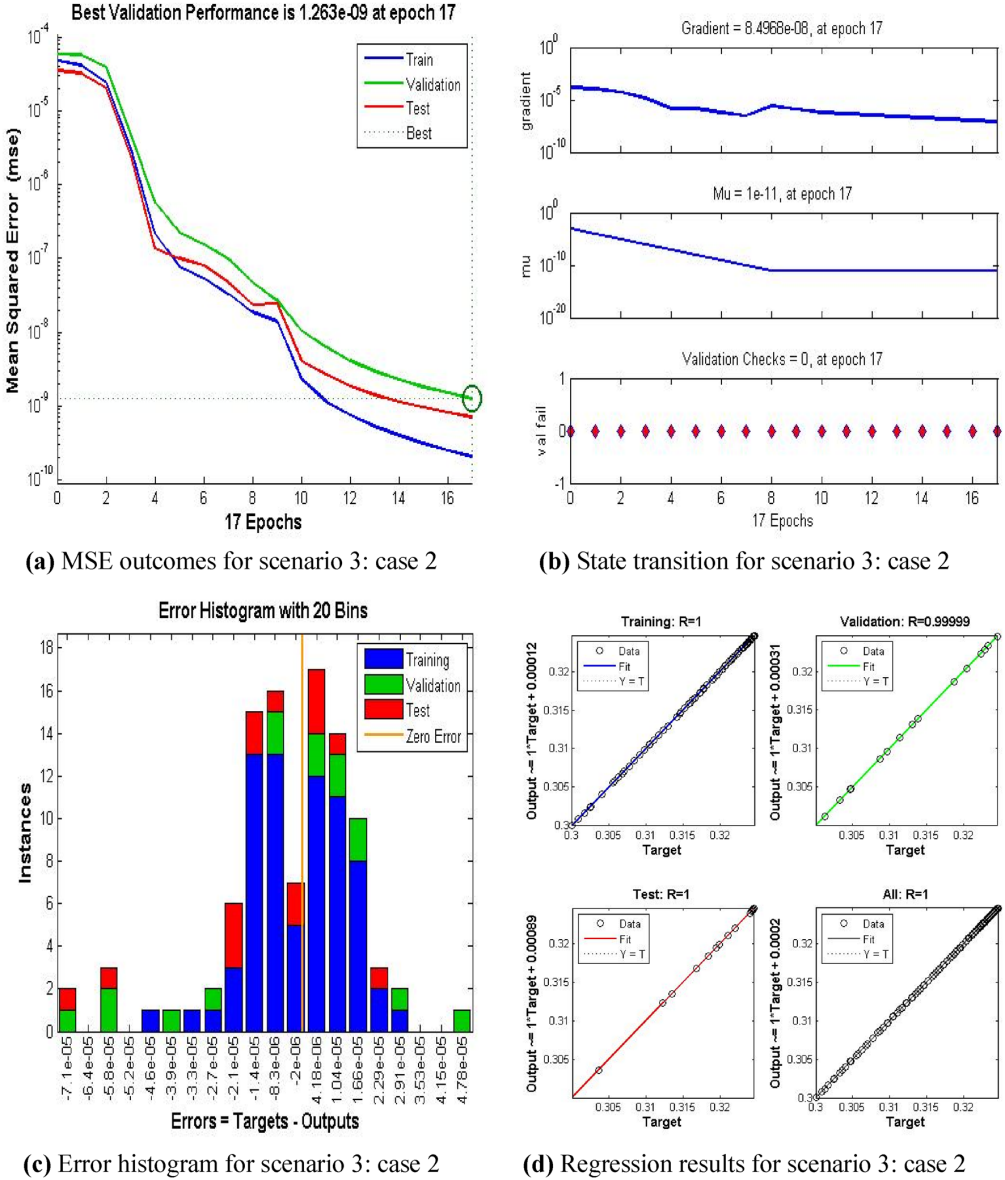
ANNs-BRS outcomes for solving the hybrid nanofluid flow model in scenario 3 case 2.

**Figure 6:**
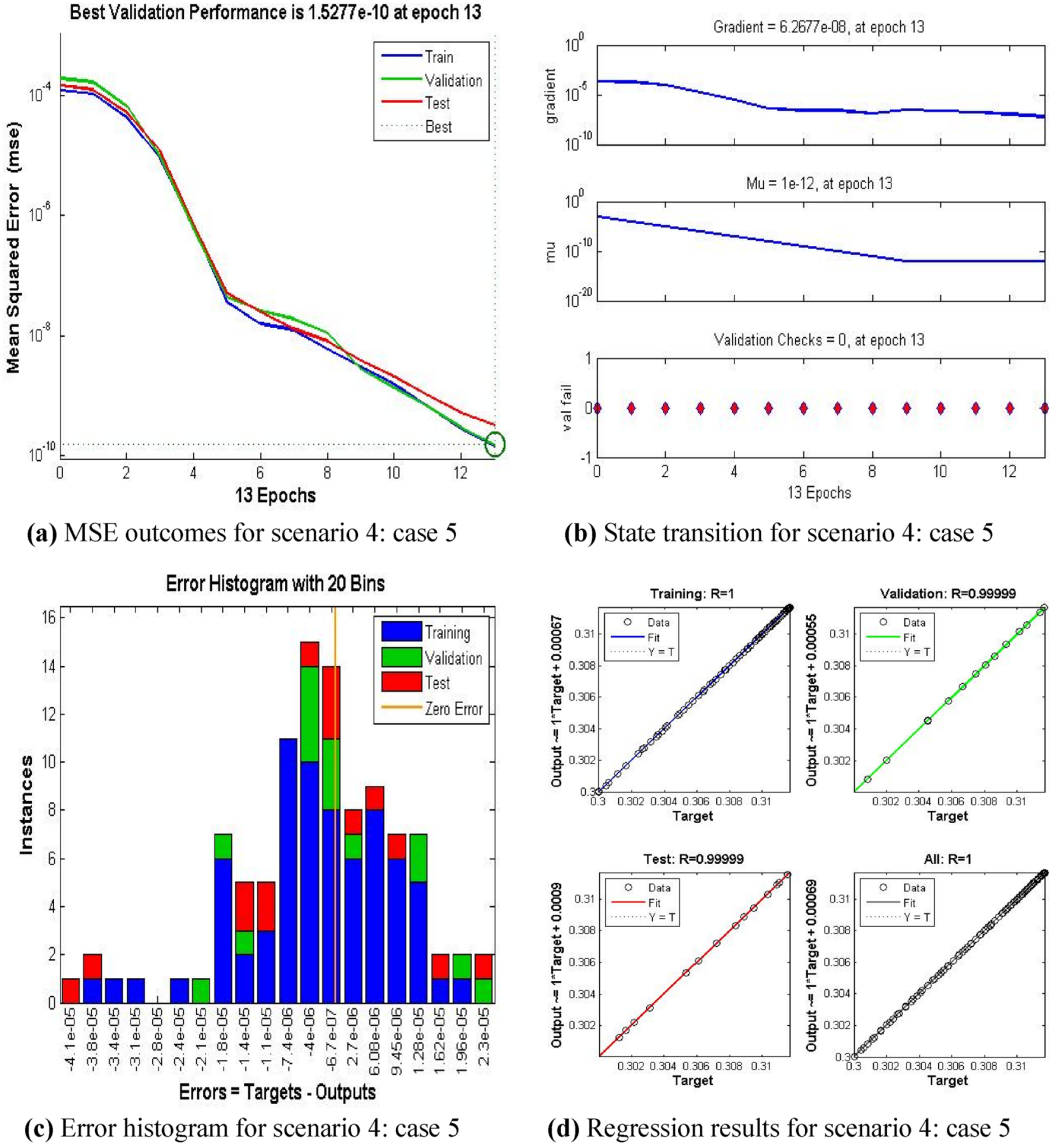
ANNs-BRS outcomes for solving the hybrid nanofluid flow model in scenario 4 case 5.

**Figure 7:**
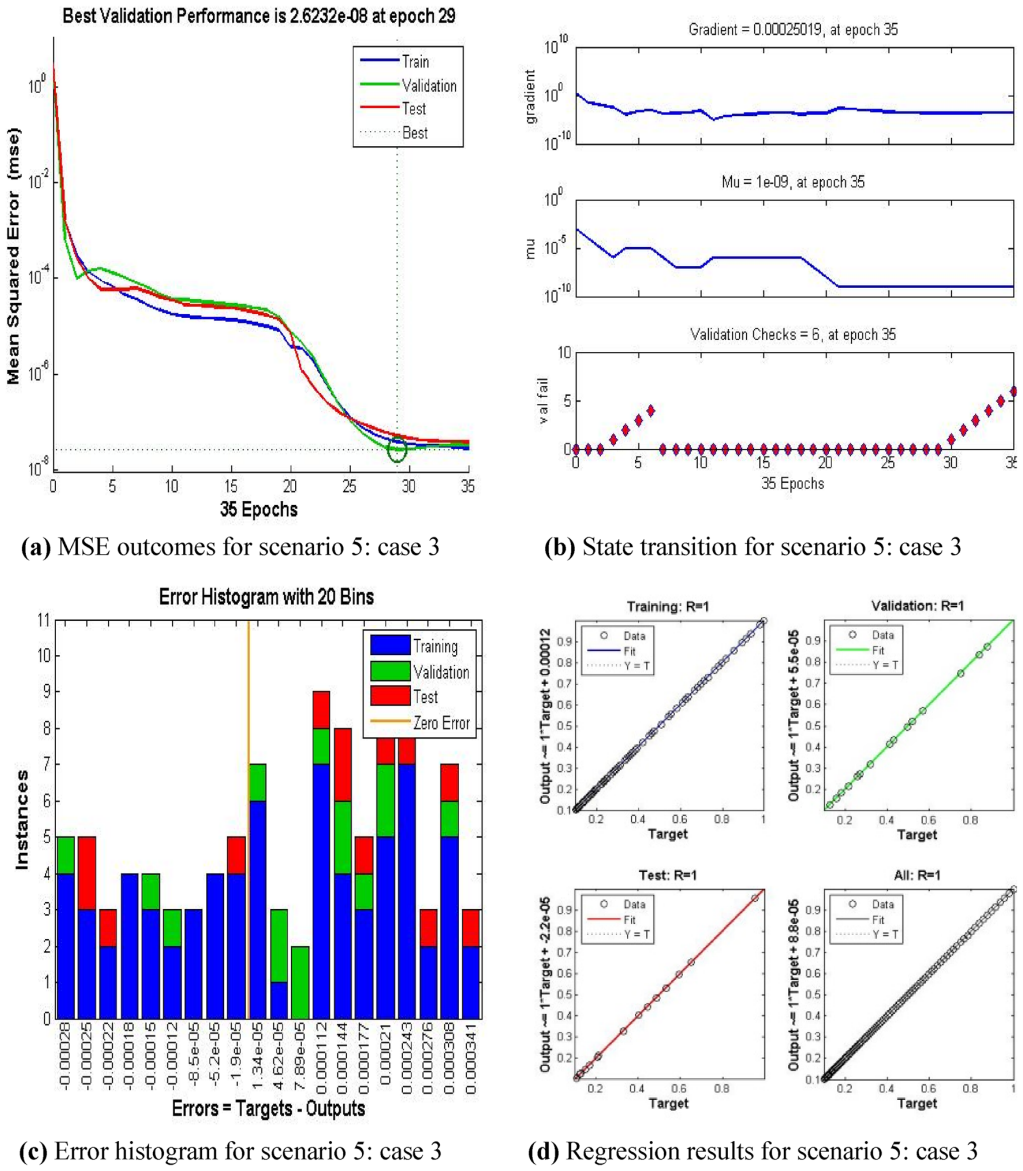
ANNs-BRS outcomes for solving the hybrid nanofluid flow model in scenario 5 case 3.

**Figure 8:**
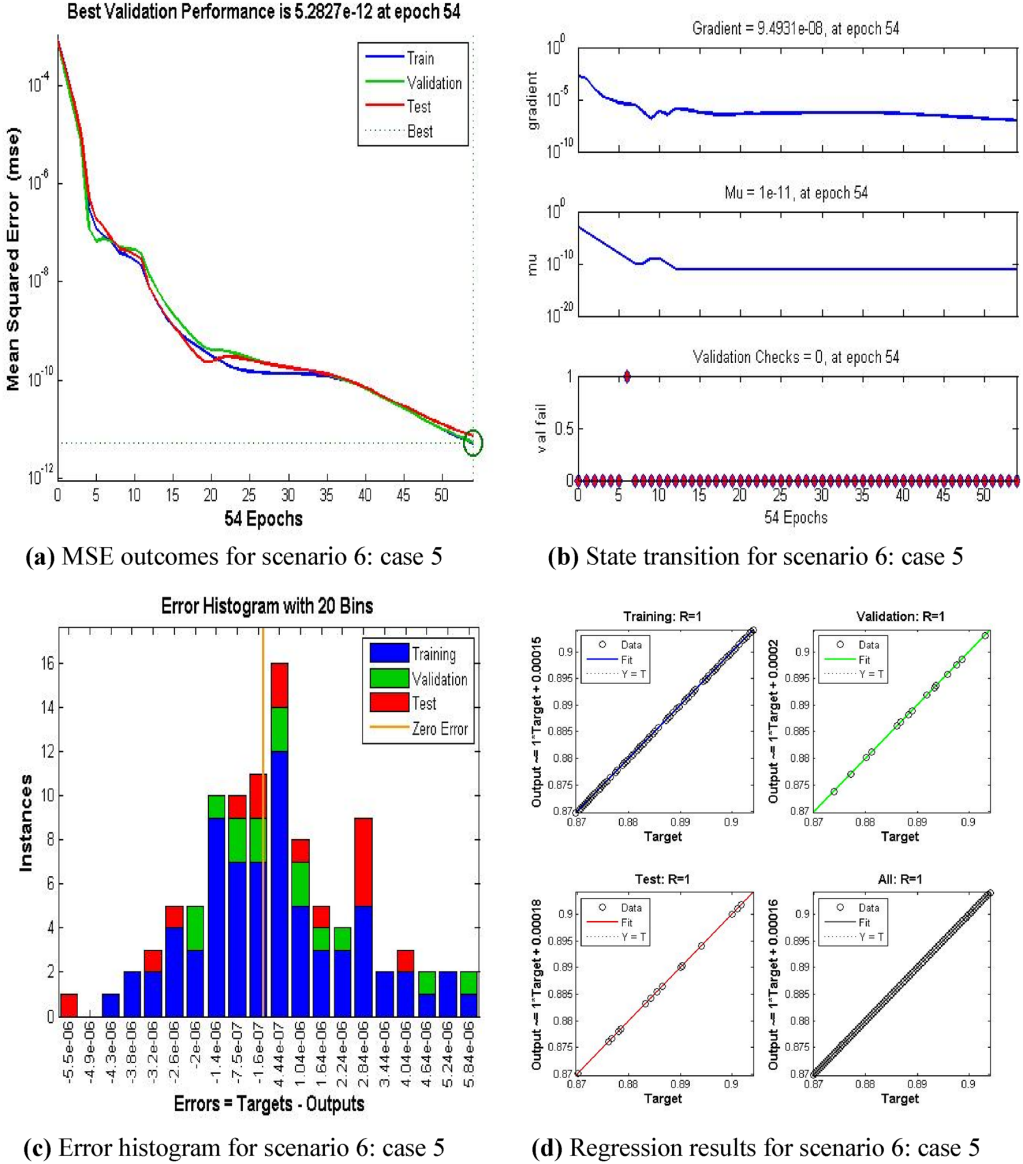
ANNs-BRS outcomes for solving the hybrid nanofluid flow model in scenario 6 case 5.

The current ANN model develop with MLP approach. MLP (Multilayer perceptron) is a fully connected multilayer neural network. It has three layers including two hidden layer and an input and output layer. The information about the MLP in the current study is given in Fig. 2. The number of the hidden neurons depend upon the size of input layer and output layer of the ANN model. The number of neurons used in current ANN model are 166. Figure 2, depicts the network architecture of neural network scheme. Figure 3a–d shows the MSE variation, state transition outcomes, error histogram plots and regression analysis namely stretching parameter $A1$. Similarly, Figs. 4a–d, 5a–d, 6a–d, 7a–d, 8a–d characterize the variation of Mean square error, state transition outcomes, plots of error histogram and regression demonstration for scenario 2 case 4, namely *A2*, Scenario 3 case 2 namely *φ*, Scenario 4 case 5 namely $F_{r}$, Scenario 5 case 3 namely $ws$ and scenario 6 case 5 namely $Q$ respectively. According to Fig. 4a–b, the best training performance is 4.935E-09 at epoch 10, with a gradient of 7.271E−08 and Mu 1E−10 at that time. Figure 3c shows the plot of error histogram, while Fig. 3d shows the regression plot for Case 3 of Scenario 1. ANNs-BRS with MSE errors in the E−09 range demonstrate the validity of the proposed method.

### Hidden layers

- A neural network with 0 hidden layer has ability to express only linearly separable functions.
- A neural network with 1 hidden layer can represent any function that consists of a continuous mapping between two finite spaces.
- A neural network with 2 hidden layer can approximate any smooth mapping to any precision and can represent any decision boundary to any accuracy using rational activation functions.
- A neural network with more than two hidden layers is usually used for deep learning models.

Using less number of neurons in the hidden layers results under fitting of the curve also using more neurons in the hidden layers results over fitting of the curve. Therefore.

- Number of neurons should be between the input and output layer.
- Number of neurons should be 0.66 size of input layer plus size of output layer.
- Number of neurons should be less than twice the size of the input layer.

From Figs. 4a, 5a, 6a, 7a, and 8a it can be observed that best training performance are [3.680E−11, 1.263 E−09, 1.527E−10, 2.623E−08, 5.287E−12] at epoch [33, 17, 13, 35, 54] for case 4 of scenario 2, case 2 of scenario 3, case 5 of scenario 4, case 3 of scenario 5 and case 5 of scenario 6 respectively. One may notice from these figures that the trained data is validated very accurately for all scenarios. Figures 4b, 5b, 6b, 7b, and 8b presents the outcomes of state transition describing gradient values, epochs numbers along with validation checks for various scenarios. From these figures one may observe that gradient values are [9.736E−08, 8.496E−08, 6.267E−08, 2.56E−3, and 9.493E−08], Mu having values [1E−11, 1E−11, 1E−12**,** 1E−09 and 1E−11] respectively, for each scenario, at epochs [33, 17, 13, 35 and 54]. The plots of error histograms are presented in Figs. 4c, 5c, 6c, 7c, and 8 for case 4 of scenario 2, case 2 of scenario 3, case 5 of scenario 4, case 3 of scenario 5 and case 5 of scenario 6 respectively. These plots indicate that the difference between target and output error is almost negligible. When the distribution of data in an error histogram is far from the zero error line, it means that the errors in the measurements are systematic and not random. Systematic errors are errors that consistently bias the results in the same direction, and are usually caused by factors such as instrument calibration errors, incorrect data processing methods, or human errors in the measurement process. The regression graphs for case 4 of scenario 2, case 2 of scenario 3, case 5 of scenario 4, case 3 of scenario 5 and case 5 of scenario 6 are shown below in, Figs. 4d, 5d, 6d, 7d, and 8d respectively. Also Figs. 4d, 5d, 6d, 7d, and 8d describes regression plots which calculates the correlation between outputs and targets, if R is close to 1 indicates close relationship while near to 0 represents random relationship. In terms of training and testing one can see that a correlation with a value close to unity provides ideal model, proving the validity of the suggested model artificial neural network with Bayesian regularization scheme (ANNs-BRS) for hybrid Nano-material carbon nanotubes based fluidic system. R is the coefficient of determination and it is calculated for linear regression and different types of statistical model also for linear regression it is calculated by using Pearson correlation coefficient (r). But for the different types of statistical model R is calculated by using regression outcomes as shown below.

- Calculating R using Pearson correlation coefficient
  $$R = r^{2}$$
- Calculating R using regression outcomes
  $$R = 1 - \frac{sum\,of\,sqaured\,residuals}{{total\,sum\,of\,squares}}$$

These are the two formulas by which we find the value of coefficient of determination also in real life examples. Also the minimum value for R is 0.01 and the larger value for R is 0.25 for better results, depending on sampling different for each case. Tables 3, 4, 5, 6, 7 and 8 are generated for Scenario (1–6) respectively. The relevant parameter values for each of the six scenarios are shown in these tables. The concept of a zero-second calculation is often used in computer science and engineering to describe extremely fast calculations that can be performed almost instantly. This can occur for several reasons, such as the use of specialized hardware, optimized algorithms, or the calculation being performed in parallel. It is important to note that the concept of a zero-second calculation is a theoretical limit, and that in practice, even the fastest calculations will take some non-zero amount of time. However, for certain applications, the time required for a calculation can be so small that it is effectively zero from a practical standpoint.

**Table 3:** Scenario 1: variation of $A1$.

Cases	MSE		Performance index	Gradient	Mu	Epoch	Time
	Training	Testing					
1	2.385E−12	3.185E−12	2.385E−12	3.42E−08	1.00E−08	22	0.00.00
2	3.514E−11	4.093E−11	3.514E−11	2.03E−09	1.00E−09	14	0.00.02
3	4.935E−09	3.823E−10	4.935E−09	7.271E−08	1.00E−10	10	0.00.00
4	4.201E−10	1.478E−10	4.201E−10	3.75E−08	1.00E−08	37	0.00.01
5	5.263E−12	2.998E−12	5.263E−12	2.37E−09	1.00E−09	51	0.00.02

**Table 4:** Scenario 2: variation of $A2$.

Cases	MSE		Performance index	Gradient	Mu	Epoch	Time
	Training	Testing					
1	2.731E−11	1.271E−12	2.731E−11	3.403E−08	1.00E−03	52	0.00.01
2	3.165E−10	4.281E−10	3.165E−10	1.952E−08	1.00E−04	66	0.00.00
3	7.481E−12	5.112E−13	7.481E−12	8.071E−08	1.00E−04	102	0.00.02
4	3.680E−11	1.248E−12	3.680E−11	9.736E−08	1.00E−11	33	0.00.00
5	3.123E−14	3.958E−12	3.123E−14	7.840E−08	1.00E−04	85	0.00.00

**Table 5:** Scenario 3: variation of $\varphi$.

Cases	MSE		Performance index	Gradient	Mu	Epoch	Time
	Training	Testing					
1	3.258E−13	1.983E−12	3.258E−13	3.156E−09	1.00E−11	52	0.00.02
2	1.263E−09	3.261E−11	1.263E−09	8.496E−08	1.00E−10	17	0.00.00
3	4.159E−12	2.131E−13	4.159E−12	7.321E−11	1.00E−11	62	0.00.00
4	3.821E−09	4.312E−10	3.821E−09	2.456E−10	1.00E−09	10	0.00.01
5	2.075E−12	5.951E−12	2.075E−12	5.639E−12	1.00E−11	111	0.00.00

**Table 6:** Scenario 4: variation of $F_{r}$.

Cases	MSE		Performance index	Gradient	Mu	Epoch	Time
	Training	Testing					
1	2.122E−13	3.244E−09	2.122E−13	5.820E−09	1.00E−11	77	0.00.02
2	5.347E−10	4.018E−13	5.347E−10	6.212E−09	1.00E−11	119	0.00.01
3	4.736E−11	5.449E−11	4.736E−11	4.213E−08	1.00E−11	17	0.00.00
4	3.112E−09	3.162E−12	3.112E−09	3.197E−08	1.00E−10	67	0.00.00
5	1.527E−10	2.022E−13	1.527E−10	6.267E−08	1.00E−12	13	0.00.00

**Table 7:** Scenario 5: variation of $ws$.

Cases	MSE		Performance index	Gradient	Mu	Epoch	Time
	Training	Testing					
1	3.258E−12	3.125E−12	3.258E−12	3.156E−09	1.00E−11	158	0.00.02
2	4.263E−09	1.231E−10	4.263E−09	8.496E−08	1.00E−10	78	0.00.00
3	2.623E−08	4.156E−13	2.623E−08	2.501E−04	1.00E−09	35	0.00.00
4	5.821E−11	5.698E−11	5.821E−11	2.456E−10	1.00E−11	47	0.00.01
5	1.983E−12	6.021E−12	1.983E−12	5.639E−12	1.00E−11	95	0.00.00

**Table 8:** Scenario 6: variation of $Q$.

Cases	MSE		Performance index	Gradient	Mu	Epoch	Time
	Training	Testing					
1	2.158E−11	1.983E−12	2.158E−11	9.256E−09	1.00E−11	252	0.00.02
2	3.463E−10	3.261E−11	3.463E−10	3.696E−08	1.00E−10	121	0.00.00
3	5.659E−12	2.131E−13	5.659E−12	5.521E−11	1.00E−11	158	0.00.00
4	4.921E−09	4.312E−10	4.921E−09	3.156E−10	1.00E−09	85	0.00.01
5	5.282E−12	5.951E−12	5.282E−12	9.493E−08	1.00E−11	54	0.00.00

For various machine learning models, the training phase might conclude at various epochs for a variety of reasons:

- *Model complexity* Because there are more parameters that need to be tuned, more complex models, like deep neural networks, often take more epochs to converge to a solution.
- *Hyper parameters* Other factors, such as the learning rate or batch size, might affect the number of epochs needed to train a model. The number of training epochs needed may vary depending on which hyper parameters are used in a given model.
- *Early stopping* Some models might use early stopping, which means that training ends when a performance parameter, like validation loss, reaches a plateau. This may cause various models to stop at various epochs.
- *Dataset* The size and complexity of the dataset can also have an impact on the number of epochs required for training. Larger and more complex datasets may require more epochs to train, as there are more examples to learn from and the models need more time to converge to a solution.

### Gradient, Mu values, MSE, best training and performance index

In ANN Gradient is defined as the derivative of a function that has more than one input variable. Which means gradient measures change in the weight function with respect to the error. In mathematics gradient is also called slope of a function which is defined as the derivative of a function at a given point. Gradient and Mu values for different parameter are shown in Tables 3, 4, 5, 6, 7 and 8. During training of the neural network we used Mu to control the Back propagation process (weight updating process). If the training stops then it means we reached the maximum value of Mu. Mean square error is the average of square of errors or it is the average square difference between output and target. The best training performance is a measure of how well a machine learning model is able to fit the training data. It is often expressed as a performance metric such as accuracy, mean squared error, F1 score, precision, recall, or others, depending on the specific goals of the system or model and the characteristics of the data being used. The formula for the best training performance will depend on the specific performance metric being used. For example, the accuracy of a binary classification model is calculated as the number of correct predictions divided by the total number of predictions. The mean squared error of a regression model is calculated as the average of the squared differences between the true values and the predicted values. The symbol for the best training performance will depend on the specific performance metric being used. For example, the accuracy of a model may be expressed as “Acc”, the mean squared error may be expressed as “MSE”, and the F1 score may be expressed as “F1”. The unit of the best training performance will also depend on the specific performance metric being used. For example, accuracy is a unitless ratio, mean squared error has units of the square of the target variable, and F1 score is unitless. The performance index is a metric used to evaluate the performance of a system, algorithm, or model. The specific definition of the performance index depends on the application, but generally it measures how well a system is achieving its goals, such as accuracy, speed, efficiency, or other desired outcomes. The calculation of the performance index can vary widely depending on the application, but common methods include using metrics such as accuracy, mean squared error, F1 score, precision, recall, and others. The choice of performance index will depend on the specific goals of the system or model, and the characteristics of the data being used.

## Result and discussions

**Figure 9:**
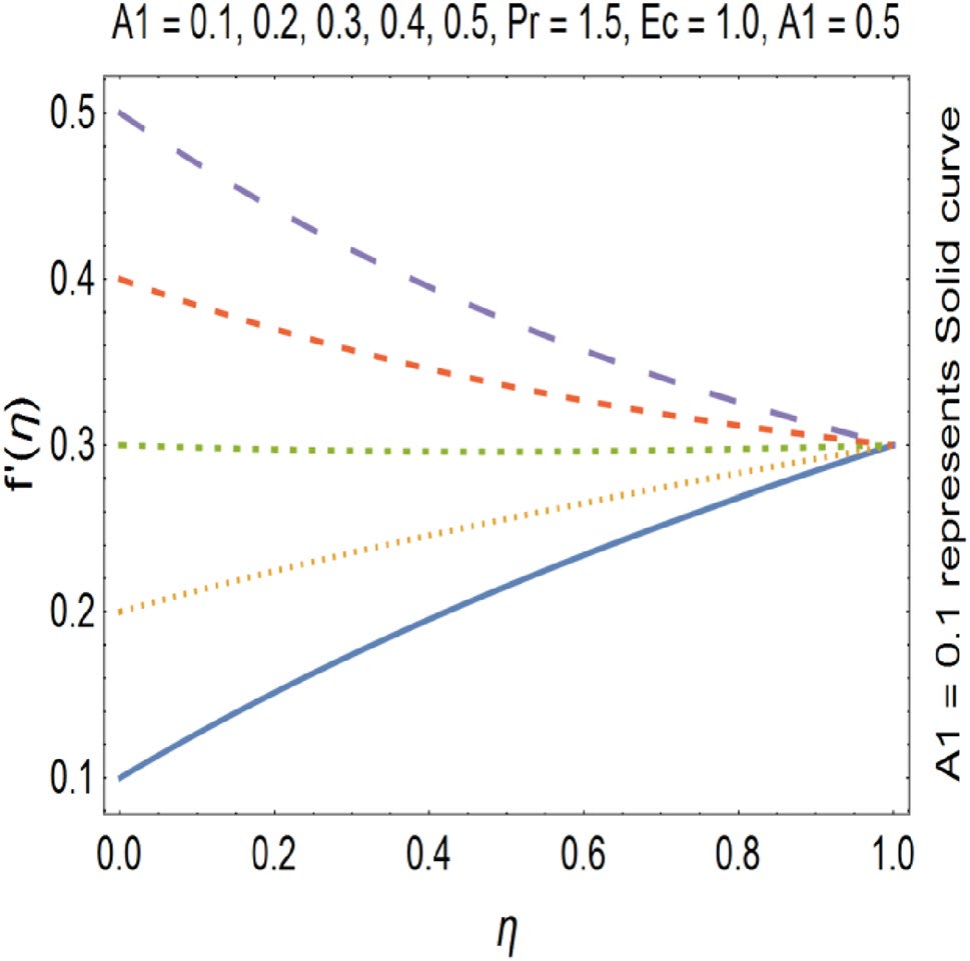
Velocity profile for various A1.

**Figure 10:**
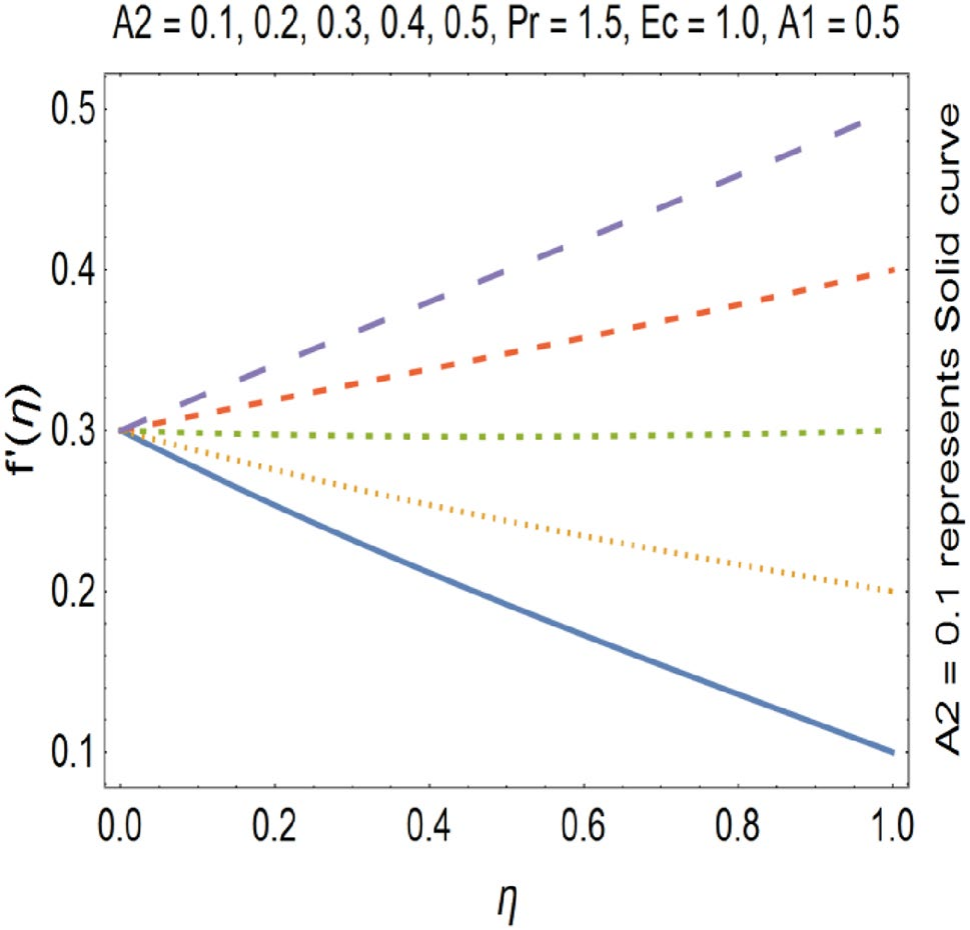
Velocity profile for various A2.

**Figure 11:**
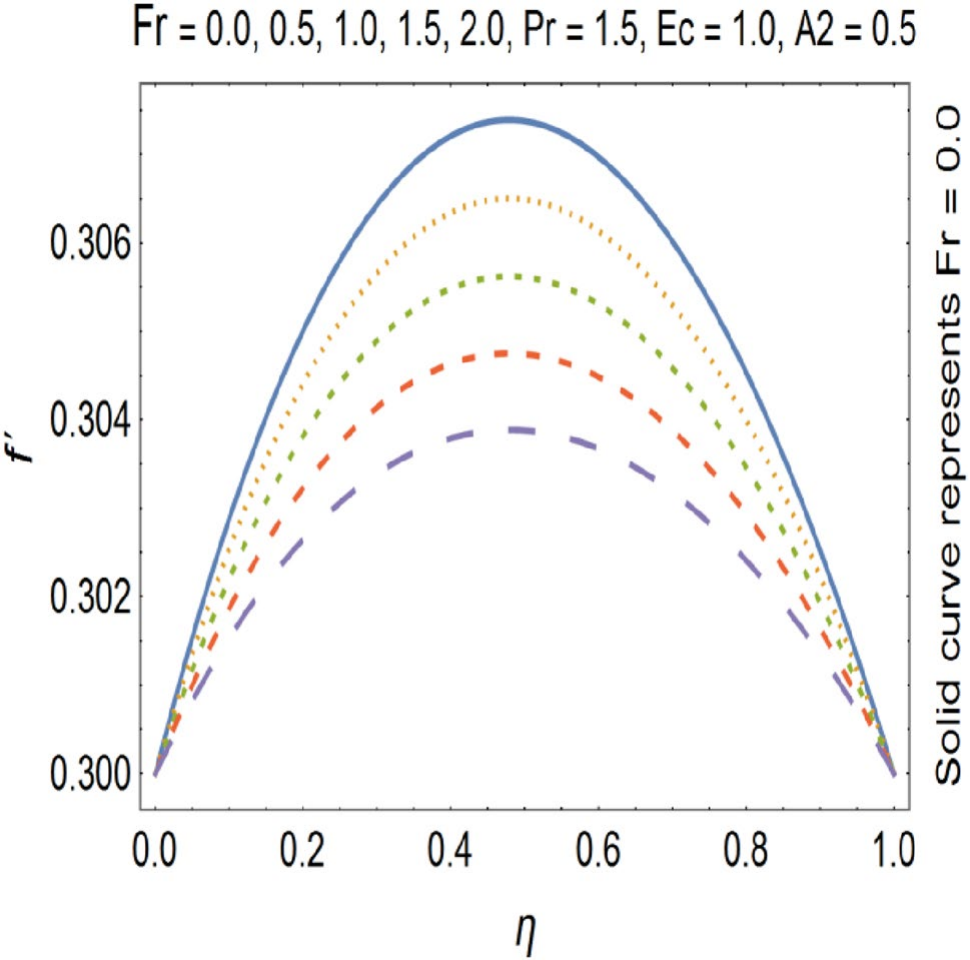
Velocity profile for various F_r_.

**Figure 12:**
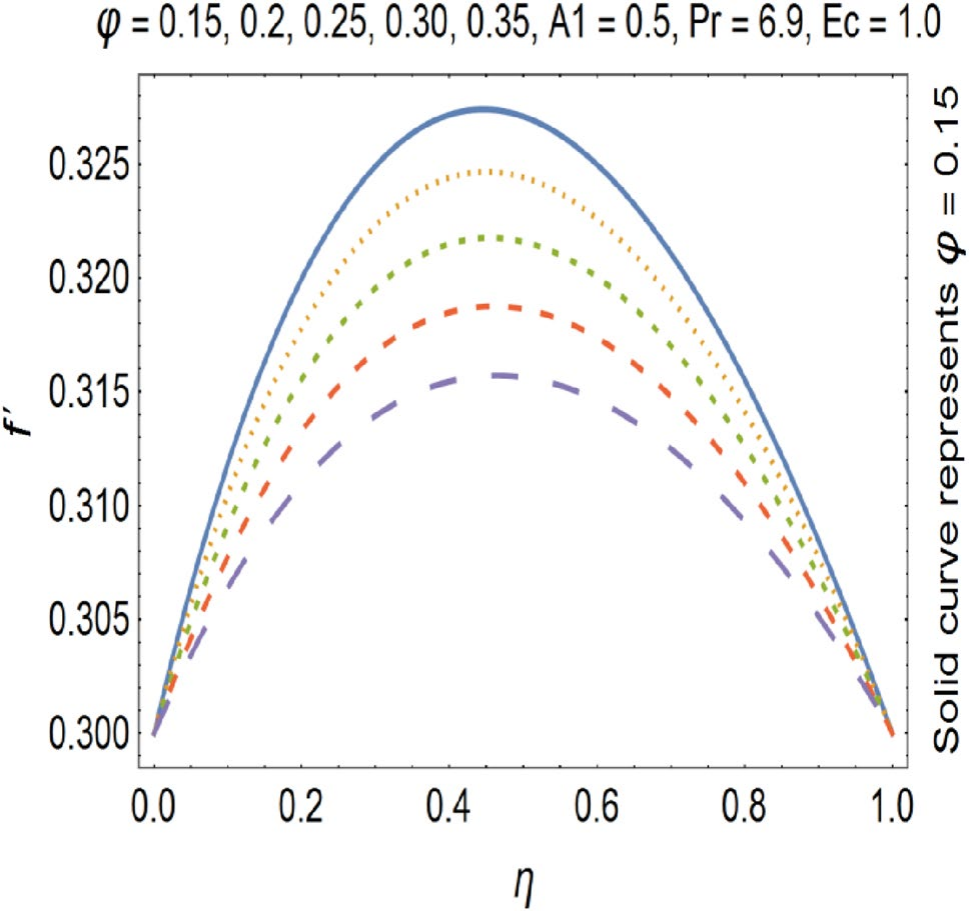
Velocity profile for various φ.

Figures 9, 10, 11 and 12 shows the effect of stretching parameters (A1, A2), Forchheimer number (Fr), Nanoparticle volume fraction (φ) respectively, on velocity profile.

**Figure 13:**
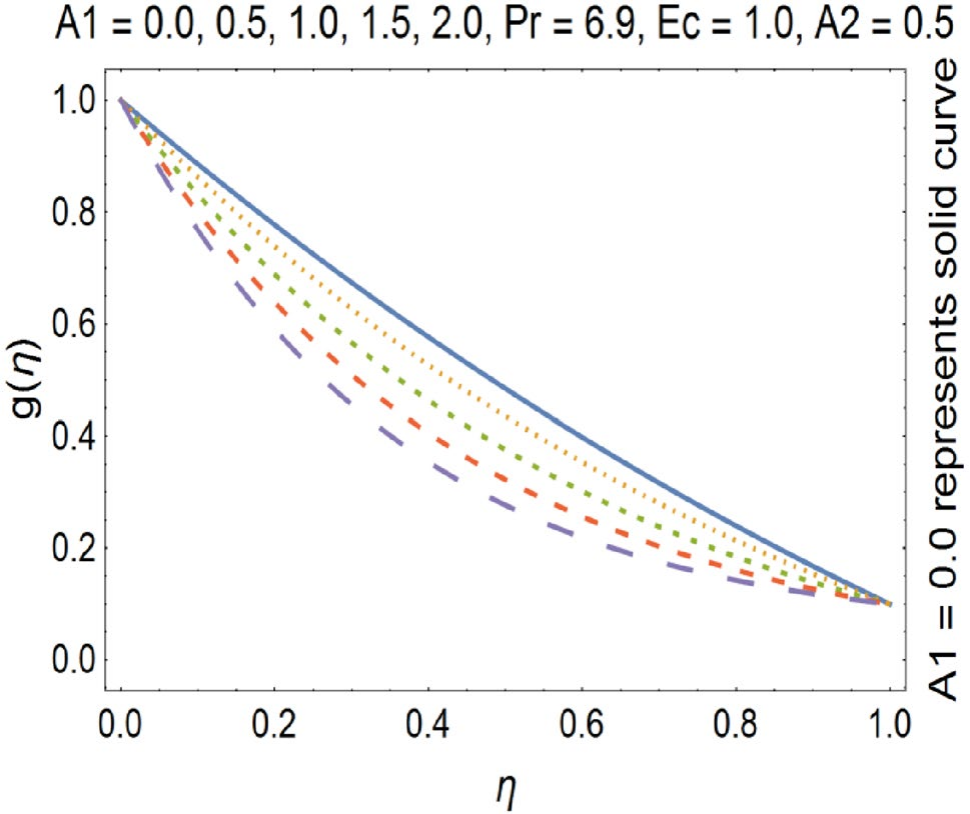
Tangential velocity profile for various A1.

**Figure 14:**
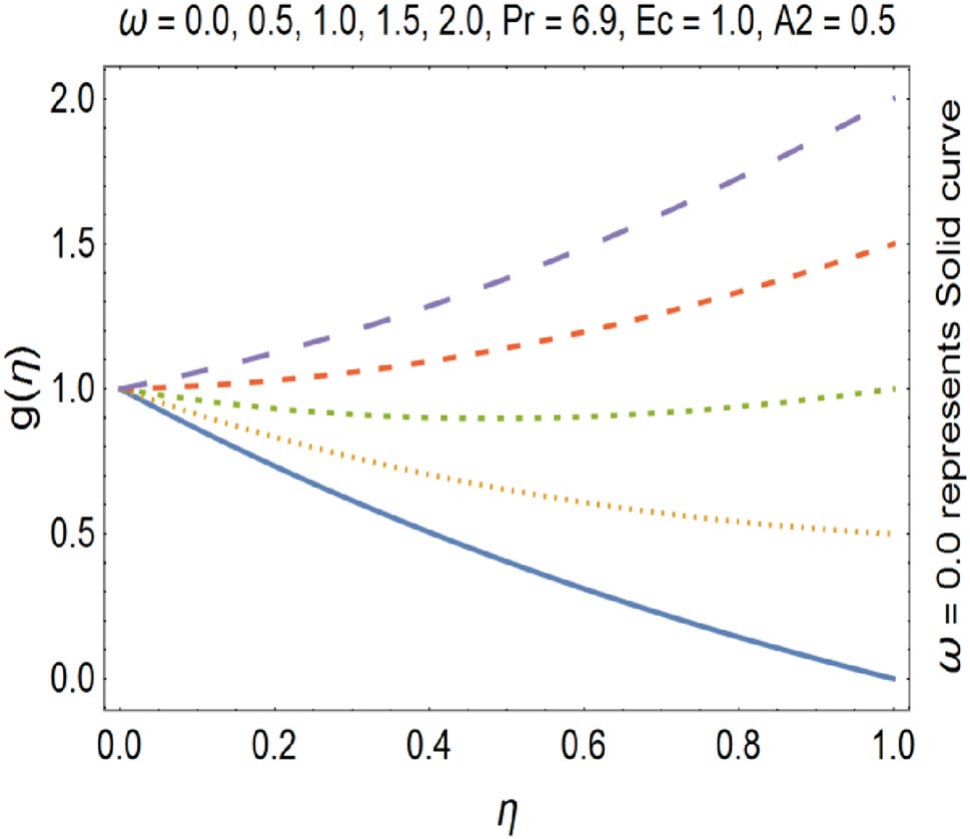
Tangential velocity profile for various ω.

SWCNT’s has higher density as compared to MWCNTs. Figures 9 and 10 shows that velocity profile gets enhanced by increasing the values stretching parameters (A1, A2).

The impact of Forchheimer number on velocity profile is depicted in Fig. 11, which shows that by increasing Forchheimer number velocity profile decreases. Figure 12 shows the influence of Nanoparticle volume fraction (φ) on velocity profile. By increasing the value of nanoparticle volume fraction (φ), density of the fluid gets increased which results a decrease in velocity of the fluid. By increasing the values of forchheimer number velocity profile gets decrease this is due to the fact that inertia of the fluid gets increase by increasing forchheimer number which causes the deceleration of momentum and velocity profile also the nano particle volume faction has direct relation with the density of the fluid so by increasing the values nano particle volume fraction the density of the fluid improves and velocity and density has inverse relation with each other. Therefor velocity of the fluid decreases by increasing nano particle volume fraction. Increase in stretching parameter causes increase in the velocity of the fluid because when we are going to increase the stretching parameter the viscosity of the fluid declines hence velocity of the resulting fluid gets increased. On the other hand by increasing the stretching parameter momentum boundary layer thickness gets reduce which also cause the increase of the velocity of the fluid.

The impact of stretching parameter A1 on g (η) is depicted in Fig. 13, which shows that stretching velocity of the fluid between the discs decreases. This is due to the fact that stretching increases in radial direction which cause the decreases in velocity of the fluid in tangential direction. Figure 14, shows the impact of rotation number (ω) on tangential velocity profile. It is clear from the Fig. 14, that by enhancing the rotation number, the tangential velocity profile gets increased. Tangential velocity which is given by

$$v_{t} = r\omega$$

In above equation we have tangential velocity on left side of equation. We observe from the above equation that tangential velocity and rotational parameter has direct relation with each other. Hence increase in rotational parameter implies increase in tangential velocity. Due to increase of the stretching parameter the momentum boundary layer thickness gets decrease which results the deceleration of the tangential velocity. Suction causes the increase in the volume flow rate of fluid. As a result the tangential velocity gets decrease.

**Figure 15:**
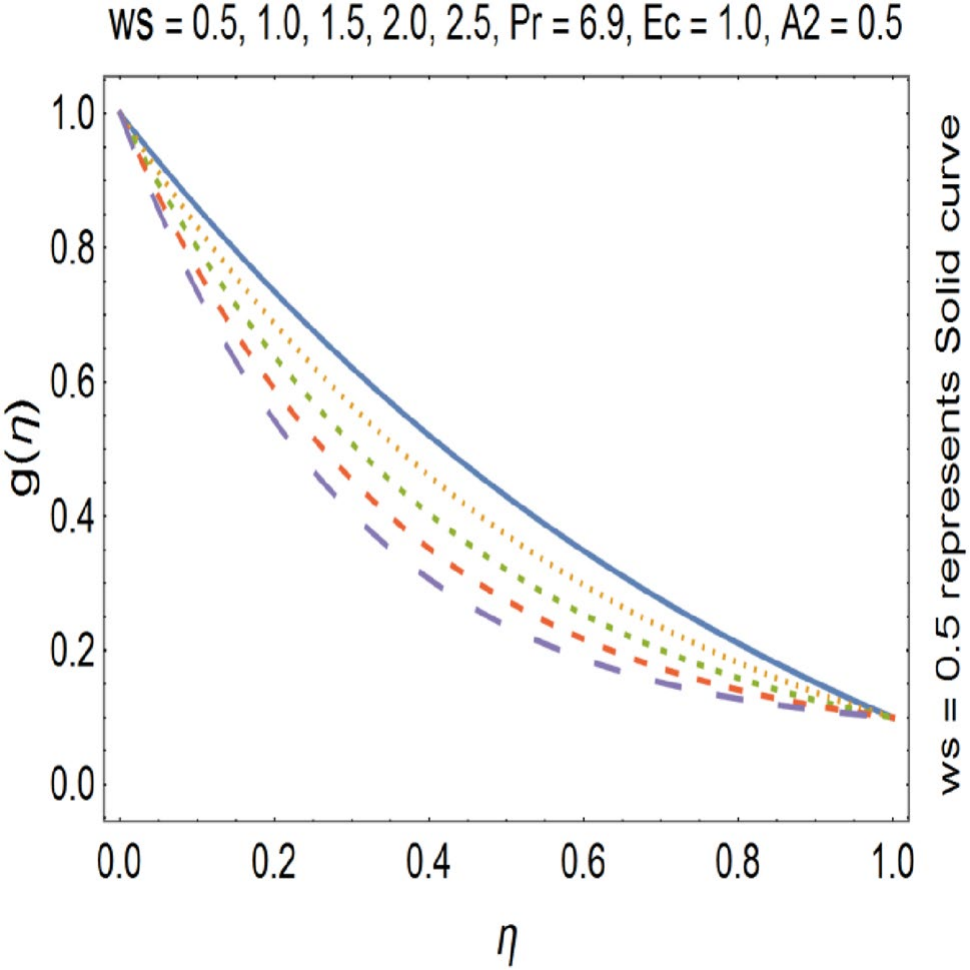
Tangential velocity profile for various ws.

**Figure 16:**
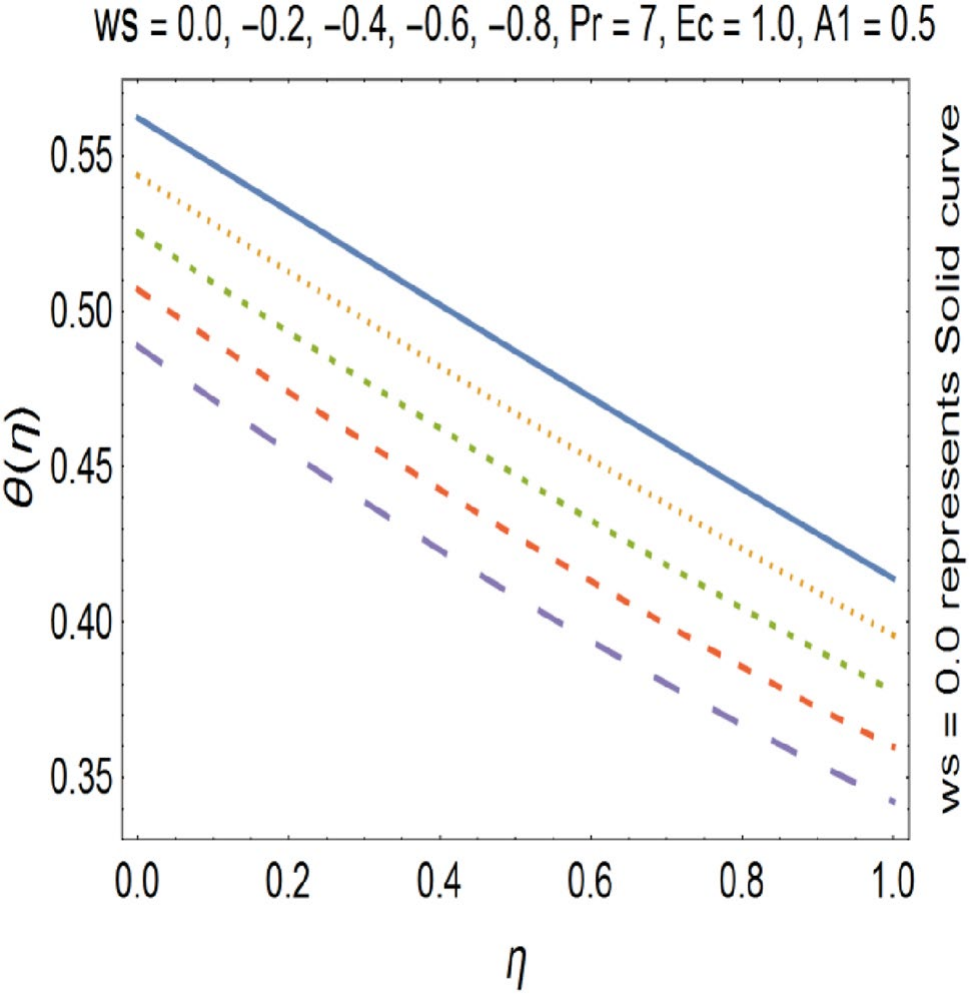
Temperature profile for various ws.

Figure 15, show the effect of suction on tangential velocity g (η). For suction we have ($ws$ > 0). In case of suction, g (η) decreases with increasing the value of suction. This is due to the fact that, increase in suction parameter causes increase in volume flow rate. Figure 16, shows the effect of injection on temperature profile (θ). For injection ($ws$ < 0), but in case of ($ws$ < 0), we see that injection and temperature profile has direct relation to each other. This is due to the fact that in case of injection volume flow rate decreases. Due to intense blowing ($ws$ < 0), speed of the fluid increases because heated liquid moves away from the stretching wall. The formula for heat source/sink parameter is shown below

$${\text{Q}} = \frac{{{\text{Q}}_{{\text{o}}} }}{{\left( {{\text{C}}_{{\text{p}}} \rho } \right)}}$$

where Q is heat source/sink parameter, Q_o_ is volumetric heat generation parameter, C_p_ is specific heat at constant pressure and *ρ* is the density of the fluid. From above formula we see that when we are going to rise the temperature of the fluid then the heat generation parameter Q_o_ increases. As a result Q increases. In case of injection the volume flow rate gets decreased. As a result the temperature of the fluid gets increase.

**Figure 17:**
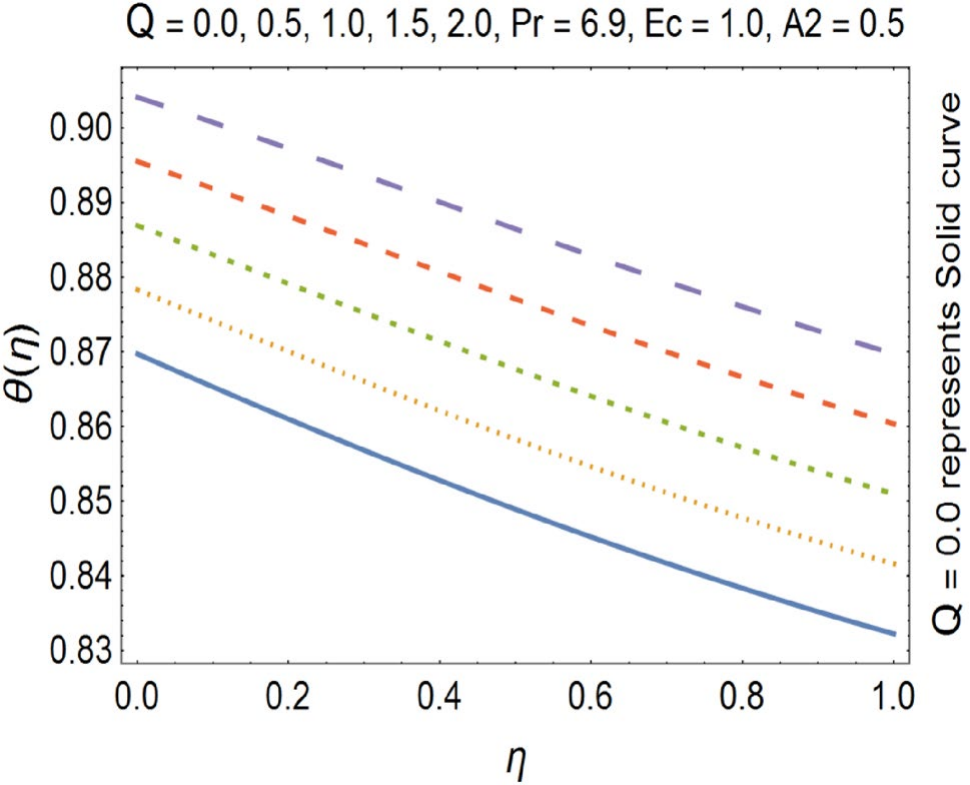
Temperature profile for various Q.

**Figure 18:**
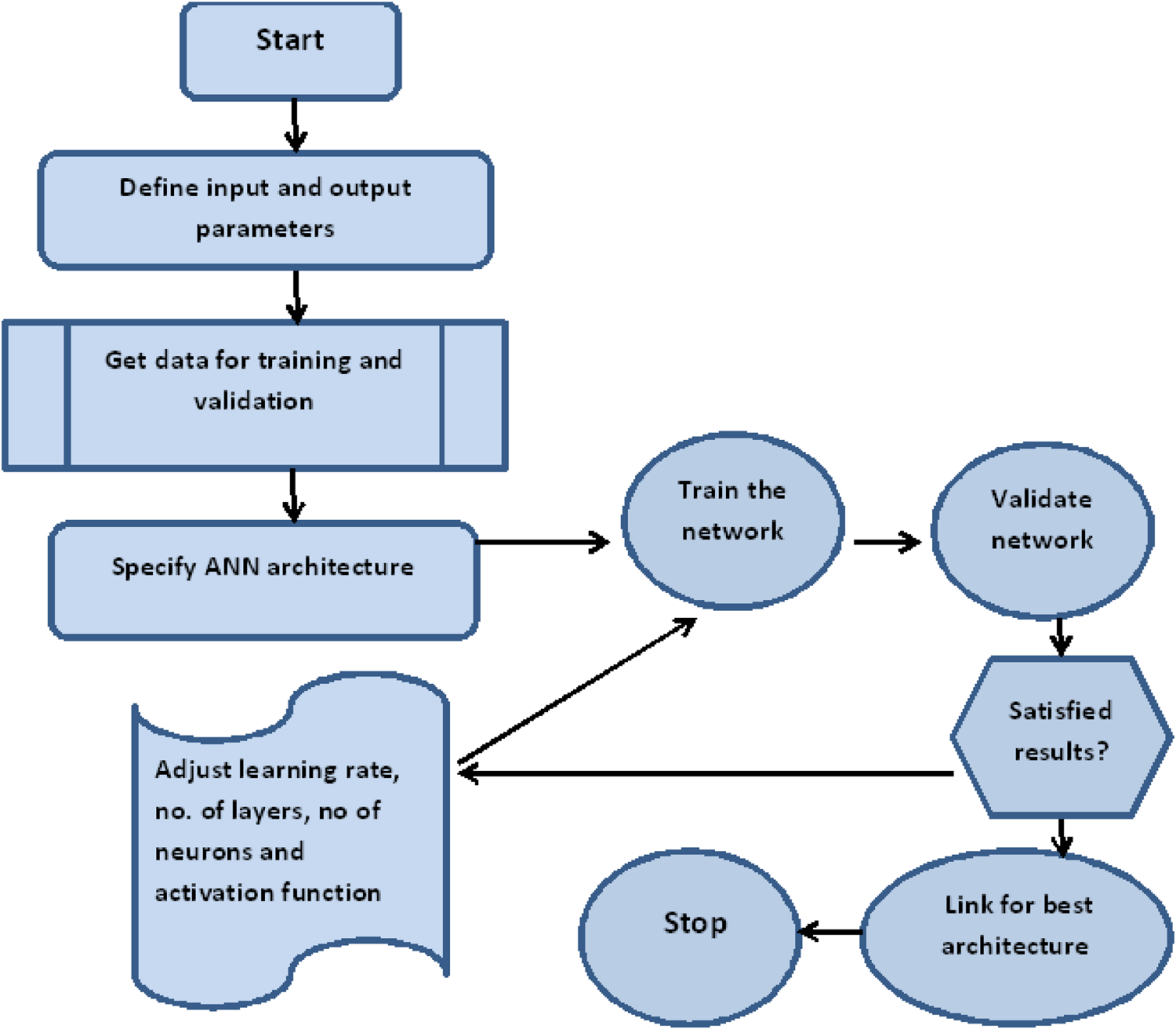
ANN training and development flow chart.

The effect of heat source/sink parameter Q on temperature profile is shown in Fig. 17. We see that by increasing the value of Q the temperature profile gets enhanced. This happens due to fact that Q and temperature profile has a direct relation to each other. The heating source can be various forms of energy such as electricity, natural gas, oil, coal, solar, etc. The heating mechanism refers to the way the heat energy is transferred to the environment. Examples include radiators, heat pumps, furnaces, boilers, etc. The flow chart for ANN development is presented in Fig. 18.

From the comparison of Tables 9, 10, we see that there is nice agreement between the values which proposed the verification of our dataset.

**Table 9:** Outcomes of skin friction coefficient for SWCNT-H_2_O at lower and upper disks.

$A1$	$A2$	$\varphi$	$F_{r}$	$ws$	$Q$	SWCNT-H_2_O^49^		SWCNT-H_2_O (our calculations)		Error	
						Re_r_cf_1_	Re_r_cf_2_	Re_r_cf_1_	Re_r_cf_2_	Re_r_cf_1_	Re_r_cf_2_
0.1	0.5	0.2	0.5	1.0	1.0	0.9196638	0.3706686	0.9196523	0.3706550	0.0000115	0.0000083
0.2	0.5	0.2	0.5	1.0	1.0	0.7202311	0.1804213	0.7202347	0.1804198	0.0000936	0.0000987
0.3	0.5	0.2	0.5	1.0	1.0	0.7969406	0.5124877	0.7969337	0.5124765	0.0000190	0.0000234
1.0	0.1	0.2	0.5	0.5	1.0	0.4569406	0.9124887	0.4569345	0.9124756	0.0000840	0.0000109
1.0	0.2	0.2	0.5	0.5	1.0	0.6721820	0.2627652	0.6721736	0.2627600	0.0000008	0.0000002
1.0	0.3	0.2	0.5	0.5	1.0	1.6602378	0.7937985	1.6602345	0.7938767	0.0000198	0.0000136
1.0	0.5	0.1	0.5	0.5	1.0	0.6560556	0.0712097	0.6560448	0.0712156	0.0000877	0.0000183
1.0	0.5	0.2	0.5	0.5	1.0	0.8194059	0.0522518	0.8194076	0.0522654	0.0000009	0.0000123
1.0	0.5	0.3	0.5	0.5	1.0	0.3591073	0.1677071	0.3591078	0.1677109	0.0000124	0.0000030
1.0	0.5	0.2	0.0	0.5	1.0	0.6543663	0.0699227	0.6543568	0.0699765	0.0000388	0.0000098
1.0	0.5	0.2	0.5	0.5	1.0	0.6554387	0.0704331	0.6554400	0.0704250	0.0000198	0.0000486
1.0	0.5	0.2	1.0	0.5	1.0	0.9196638	0.3706686	0.9196600	0.3706567	0.0000091	0.0000106
1.0	0.5	0.2	0.5	0.5	1.0	1.0850795	0.3016115	1.0850767	0.3016988	0.0000654	0.0000090
1.0	0.5	0.2	0.5	0.5	1.0	0.6166419	0.0594862	0.6166375	0.0594798	0.0000983	0.0000130
1.0	0.5	0.2	0.5	1.0	1.0	0.6166419	0.0594862	0.6166466	0.0594759	0.0000937	0.0000187
1.0	0.5	0.2	0.5	0.5	0.0	0.5572981	0.0112209	0.5572987	0.0112129	0.0000287	0.0000109
1.0	0.5	0.2	0.5	0.5	0.5	0.5708473	0.0599123	0.5708456	0.0599150	0.0005566	0.0000175
1.0	0.5	0.2	0.5	0.5	1.0	0.6127332	0.0487107	0.6127290	0.0487167	0.0000876	0.0000092

**Table 10:** Outcomes of skin friction coefficient for MWCNT-H_2_O at lower and upper disks.

$A1$	$A2$	$\varphi$	$F_{r}$	$ws$	$Q$	MWCNT-H_2_O^49^		MWCNT-H_2_O (our calculations)		Error	
						Re_r_cf_1_	Re_r_cf_2_	Re_r_cf_1_	Re_r_cf_2_	Re_r_cf_1_	Re_r_cf_2_
0.1	0.5	0.2	0.5	1.0	1.0	0.8928351	0.3856347	0.8928200	0.3856786	0.0000135	0.0000209
0.2	0.5	0.2	0.5	1.0	1.0	0.6947095	0.1877135	0.6947134	0.1877150	0.0000156	0.0000253
0.3	0.5	0.2	0.5	1.0	1.0	0.7853027	0.5114352	0.7853090	0.5114465	0.0000201	0.0000198
1.0	0.1	0.2	0.5	0.5	1.0	0.7765783	0.4567776	0.7765800	0.4567675	0.0000109	0.0000109
1.0	0.2	0.2	0.5	0.5	1.0	0.6545437	0.2580787	0.6545876	0.2580667	0.0000176	0.0000298
1.0	0.3	0.2	0.5	0.5	1.0	1.6255913	0.8184235	1.6255986	0.8184345	0.0000174	0.0000093
1.0	0.5	0.1	0.5	0.5	1.0	0.6314385	0.0540316	0.6314397	0.0540267	0.0000195	0.0000136
1.0	0.5	0.2	0.5	0.5	1.0	0.7709482	0.0207808	0.7709450	0.0207750	0.0000284	0.0000148
1.0	0.5	0.3	0.5	0.5	1.0	0.3551200	0.1730186	0.3551187	0.1730190	0.0000687	0.0000185
1.0	0.5	0.2	0.0	0.5	1.0	0.6301467	0.0540681	0.6301509	0.0540651	0.0000213	0.0000946
1.0	0.5	0.2	0.5	0.5	1.0	0.6309619	0.0536555	0.6309599	0.0536487	0.0000098	0.0000696
1.0	0.5	0.2	1.0	0.5	1.0	0.8928351	0.3856347	0.8928298	0.3854267	0.0000083	0.0000988
1.0	0.5	0.2	0.5	0.5	1.0	1.0564875	0.3217612	1.0564850	0.3217565	0.0000109	0.0000756
1.0	0.5	0.2	0.5	0.5	1.0	0.5959933	0.0422747	0.5959933	0.0422876	0.0000199	0.0000234
1.0	0.5	0.2	0.5	1.0	1.0	0.6076435	0.0463330	0.6076345	0.0463376	0.0000196	0.0000019
1.0	0.5	0.2	0.5	0.5	0.0	0.5389168	0.0172513	0.5389213	0.0172648	0.0000144	0.0000029
1.0	0.5	0.2	0.5	0.5	0.5	0.5449527	0.0581194	0.5449587	0.0581234	0.0000106	0.0000382
1.0	0.5	0.2	0.5	0.5	1.0	0.5874836	0.0336106	0.5874908	0.0336234	0.0000177	0.0000109

### Margin of error

**Figure 19:**
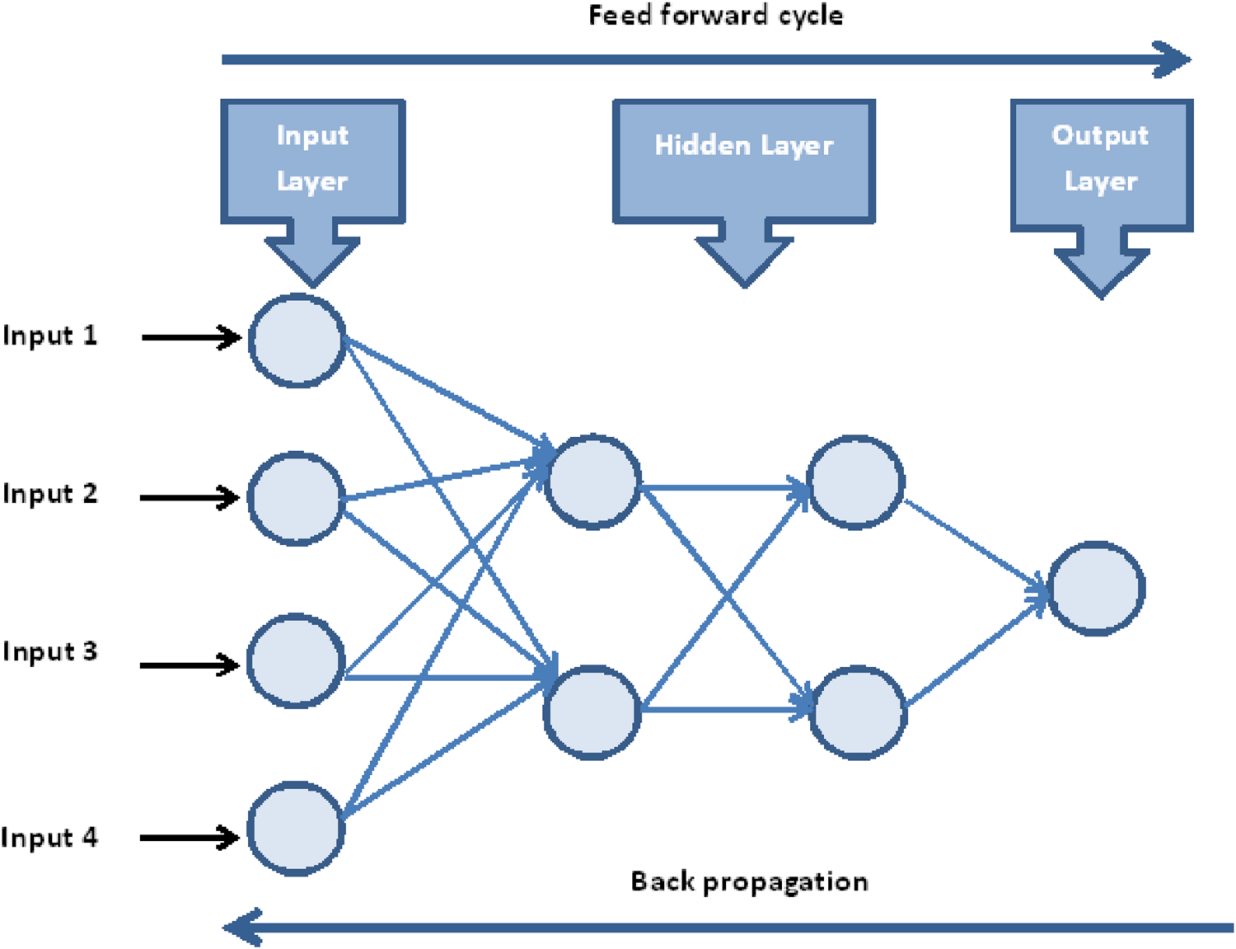
Configuration topology for MLP network.

Margin of error is range of values that differ from the actual value. It can be calculated by using the following formula

$${\text{Margin}}\,{\text{of}}\,{\text{error}}\, = \,\frac{{z^{*} \sqrt {(p^{*} (1 - p))} }}{\sqrt n }$$

here $z^{*}$ is desired confidence level, *p* is sample population and *n* is sample size. The values of margin of error for skin friction coefficient for both SWCNT-H_2_O and MWCNT-H_2_O at lower and upper disks are presented in Table 11 (Fig. 19).

**Table 11:** Margin of error.

SWCNT-H_2_O		MWCNT-H_2_O	
Re_r_cf_1_	Re_r_cf_2_	Re_r_cf_1_	Re_r_cf_2_
0.0000115	0.0000083	0.0000135	0.0000209
0.0000936	0.0000987	0.0000156	0.0000253
0.0000190	0.0000234	0.0000201	0.0000198
0.0000840	0.0000109	0.0000109	0.0000109
0.0000008	0.0000002	0.0000176	0.0000298
0.0000198	0.0000136	0.0000174	0.0000093
0.0000877	0.0000183	0.0000195	0.0000136
0.0000009	0.0000123	0.0000284	0.0000148
0.0000124	0.0000030	0.0000687	0.0000185
0.0000388	0.0000098	0.0000213	0.0000946
0.0000198	0.0000486	0.0000098	0.0000696
0.0000091	0.0000106	0.0000083	0.0000988
0.0000654	0.0000090	0.0000109	0.0000756
0.0000983	0.0000130	0.0000199	0.0000234
0.0000937	0.0000187	0.0000196	0.0000019
0.0000287	0.0000109	0.0000144	0.0000029
0.0005566	0.0000175	0.0000106	0.0000382
0.0000876	0.0000092	0.0000177	0.0000109

## Conclusions

The hybrid Nano-material of carbon nanotubes (CNT’s) based fluidic system is investigated in this paper using an artificial neural network with Bayesian regularization scheme (ANNs-BRS). Following are the main goal of this study.

1. Between two stretchable rotating disks the Darcy Forchheimer flow of carbon nanotubes was investigated.
2. The obtained PDE’s are converted into ODE’s by using similarity transformation.
3. We observe that inertia of the fluid gets increase by increasing forchheimer number. which causes the deceleration of momentum and velocity profile, Also by increasing the nanoparticle volume fraction ($\varphi$) density of the fluid will gets increase, which results the decrease in velocity profile. Increase in stretching parameters ($A1,\,A2$) causes increase in velocity profile because increase in stretching parameter results decrease in viscosity.
4. Due to direct relation *v*_*t*_ = *rω* the tangential velocity profile (g(η)) increases by increasing the values of rotation number (ω).
5. Increase in stretching parameter ($A1$), the momentum boundary layer thickness gets decrease which results the deceleration of the tangential velocity.
6. Suction ($ws$ > 0) causes the increase in the volume flow rate of fluid. As a result the tangential velocity gets decrease.
7. Temperature profile depicts increasing behavior for heat source/sink parameter (Q) and injection ($ws$ < 0).

## Data Availability

The datasets used and/or analyzed during the current study available from the corresponding author (A. A.) on reasonable request.

## List of symbols

$A_{1} ,A_{2}$: Stretching parameter of lower and upper disks
$$B_{o}$$: Magnetic field
$C_{f2} ,C_{f1}$: In lower and upper disks Skin friction coefficient
$C_{f}$: Skin friction coefficient
$$E_{c}$$: Eckert number
$$F_{r}$$: Forchheimer coefficient
$F$: Local inertia coefficient
$h_{1} ,h_{2} \,$: Local transfer coefficients
$$Ha$$: Hartmann number
$K$: Effective thermal conductivity
***K***: Permeability
$K^{*}$: Rosseland mean absorption coefficient
$Nu_{1} ,Nu_{2}$: Nusselt number for lower and upper disks
$$P_{r}$$: Prandtl number
$q_{r}$: Heat flux (Radiative)
$q_{w}$: Heat flux (Wall)
$$R_{d}$$: Radiation parameter
$R_{e}$: Dimensionless Reynolds number
$T_{1} ,T_{2}$: Temperature at lower and upper disks
$$u,v,w$$: Velocity components
$$r,\theta ,z$$: Cylindrical coordinates
$ws$: Suction/ injection parameter
Cp: Thermal Capacity
ANN: Artificial Neural Network
$ws$: Suction/ injection parameter

## Greek symbols

$$\alpha_{1} ,\alpha_{2}$$: Stretching rates
$$\gamma_{1} ,\gamma_{2}$$: Biot numbers
$$\lambda$$: Porosity parameters
$\mu$: Effective dynamic viscosity
$\delta^{*}$: Stephen-Boltzmann constant
$$\sigma$$: Electrical conductivity
$$\tau_{w}$$: Total stress
$\tau_{z\,r} ,\,\tau_{z\theta }$: Lower rotating disk Shear stresses
$\varphi$: Nanoparticle volume fraction
$$\omega_{1}$$: Rotational parameter
$$\omega_{1} \,,\omega_{2}$$: Angular velocities
$\eta$: Dimensionless similarity variable
MSE: Mean square error
BRs: Bayesian regularization scheme
ODE: Ordinary differential equation
PDE: Partial differential equation

## Subscripts

$$f$$: Fluid
$$N_{f}$$: Nanofluid
$$CNT$$: Carbon nanotube

## Superscript

$Q$: Heat source/sink parameter
